# Supplementary material for: An in silico and in vitro human neuronal network model reveals cellular mechanisms beyond NaV1.1 underlying Dravet syndrome
Source: Stem Cell Reports. 2023 Jul 6;18(8):1686–700. doi: 10.1016/j.stemcr.2023.06.003 (PMC10444571; doi:10.1016/j.stemcr.2023.06.003)
Supplement: Document S1. Supplemental experimental procedures and Figures S1–S4 [file mmc1.pdf]

Stem Cell Reports, Volume *18*

Supplemental Information

An *in silico* and *in vitro* human neuronal network model reveals cellular mechanisms beyond $Na_V1.1$ underlying Dravet syndrome

Nina Doorn, Eline J.H. van Hugte, Ummi Ciptasari, Annika Mordelt, Hil G.E. Meijer, Dirk Schubert, Monica Frega, Nael Nadif Kasri, and Michel J.A.M. van Putten

Supplemental Figures

A

Parameter	*Modulated part of NaV1.1*	*Effect of increasing value*
γ_{Na}	Maximal Conductance	Hyperactive neurons
$\gamma_{\tau m}$	Both rate constants m-gate	Hyperactive neurons
$\gamma_{\tau h}$	Both rate constants h-gate	Hyperactive neurons
$\gamma_{\alpha m}$	Activation rate constant m-gate	Hyperactive neurons
$\gamma_{\beta m}$	Deactivation rate constant m-gate	Hypoactive neurons
$\gamma_{\alpha h}$	Activation rate constant h-gate	Hyperactive neurons
$\gamma_{\beta h}$	Deactivation rate constant h-gate	No effect
ΔV_m	Shift voltage sensitivity m-gate	Hypoactive neurons
ΔV_h	Shift voltage sensitivity h-gate	Hyperactive neurons
γ_{NaP}	Fraction of persistent sodium channels	Hyperactive neurons

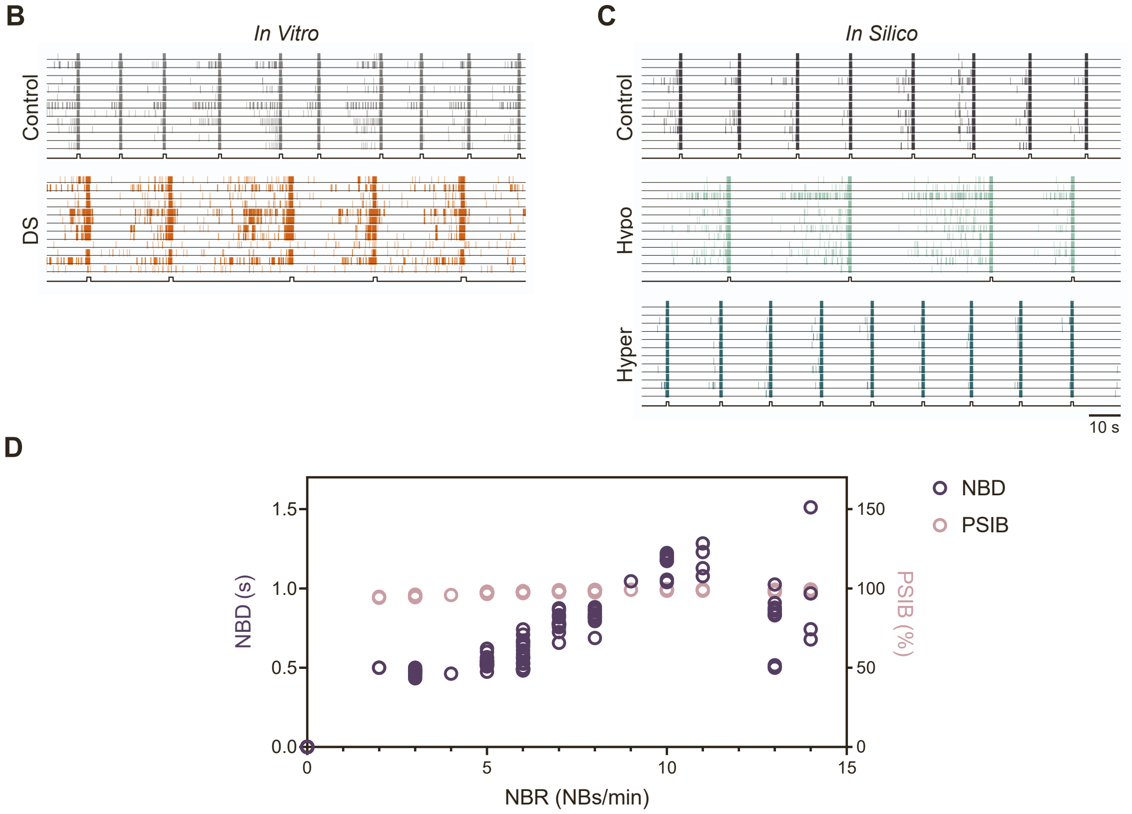

Figure S1: The effect of sodium channel modifications on the *in silico* neuron and network activity.
A) The effect of increasing the different modification parameter values (supplemental equations 1-5) on the excitability of the neuron. **B)** Representative raster plots showing 100 seconds of spontaneous activity of control and DS networks *in vitro*, with black lines indicating detected NBs. **C)** Representative raster plots showing simulations with the control *in silico* model as it is and when sodium channel modifications are made that result in hypoexcitable neurons (hypo) and hyperexcitable neurons (hyper). **D)** Quantification of the Network Burst Rate (NBR), Network Burst Dutation (NBD), and Percentage of Spikes in Network Bursts (PSIB) of the parameter space exploration with sodium channel modifications. NBD and PSIB are reported against the NBR from the corresponding simulations. The NBD in control never exceeded the NBD in DS networks with low NBR values, and the PSIB never decreased.

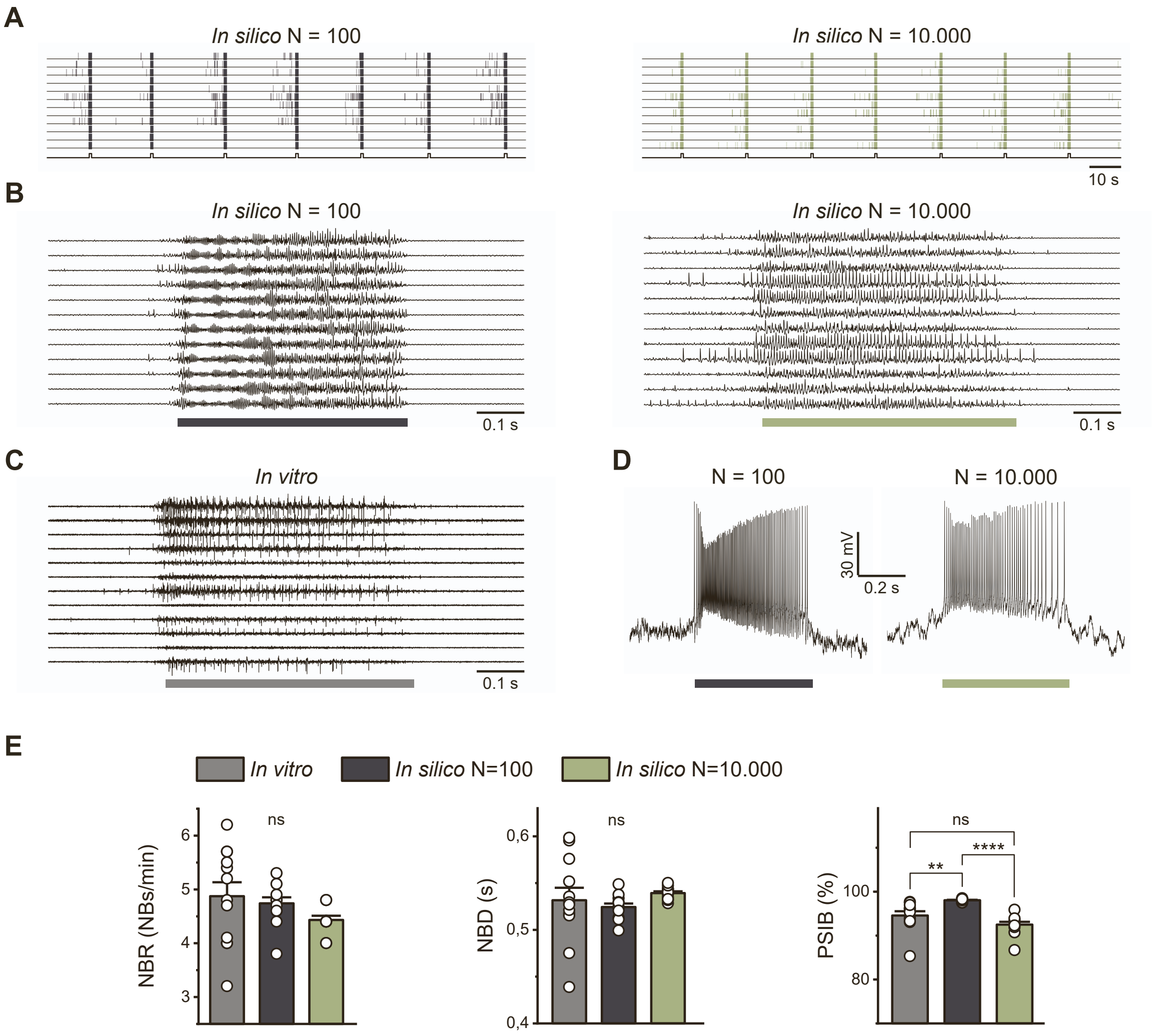

Figure S2: *In silico* simulations with 10.000 neurons result in a PSIB more comparable to control *in vitro* observations

A) Representative raster plots showing 100 seconds of simulated activity from the control *in silico* model with 100 neurons (left) and with 10.000 neurons (right). **B)** Voltage recordings from the virtual MEA electrodes showing an NB (marked below) in a simulation with 100 neurons (left) and with 10.000 neurons (right). **C)** Voltage recording from MEA electrodes showing an NB in an *in vitro* control network. **D)** The membrane potential of single neurons during an NB in a network simulation with 100 neurons (left) and 10.000 neurons (right). Due to the high synaptic input, neurons in the 100-neuron network go into depolarization block, resulting in MEA recordings with low-amplitude voltage fluctuations at the start of an NB. **E)** Quantification of Network Burst Rate (NBR), Network Burst Duration (NBD), and Percentage of Spikes in Network Bursts (PSIB) for 12 wells *in vitro* (600 s recordings) and 12 simulated networks per model *in silico* (600 s simulations with N=100 networks, and 150 s simulations with N=10.000 networks). Data represent mean $\pm$ SEM, ns $P>0.05$, ** $P<0.005$, **** $P<0.0001$. Means were compared with a two-way ANOVA with Bonferroni correction for multiple testing.

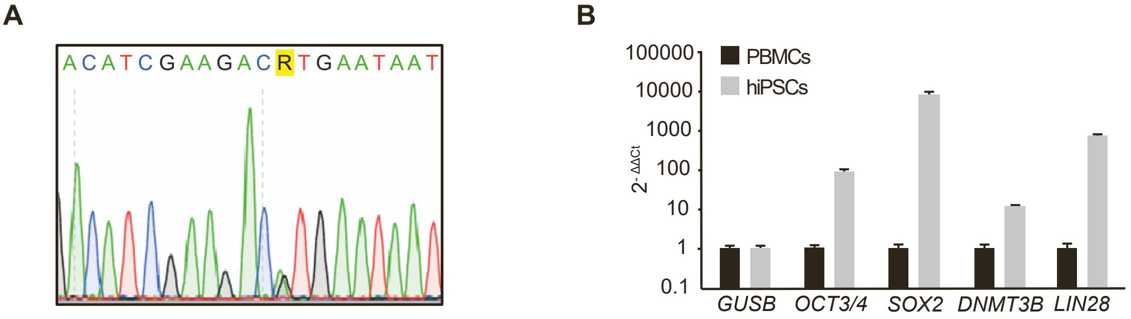

Figure S3: Mutation verification and pluripotency quantification.
A) Chromatogram of sequencing results depicting the heterozygous missense mutation c.4168G>A p.Val1390Met in *SCN1A* B) Quantitative real time PCR of pluripotentcy markers in hiPSCs relative to PBMC (perepheral mononuclear blood cells). Delta ct levels of octamer-binding transcription factor 3/4 (*OCT3/4*), SRY-box 2 (*SOX2*), *DNMT3B*, and *LIN28*, using glucuronidase beta (*GUSB*) as housekeeping gene, displayed as the relative gene expression normalized to *GUSB* levels.

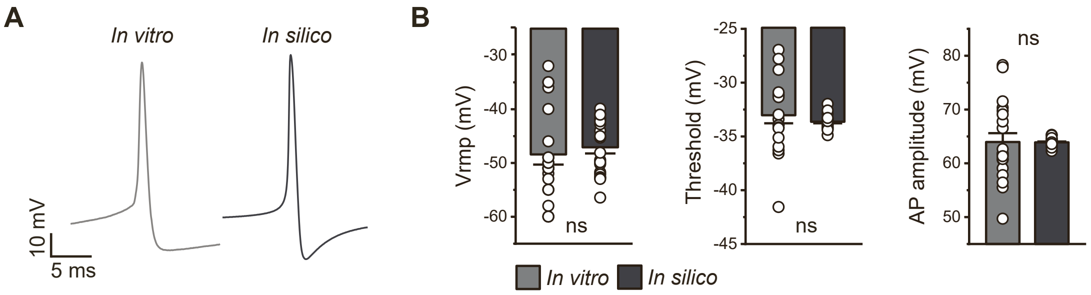

Figure S4: Comparison of *in vitro* and *in silico* intracellular AP waveshapes.
A) Representative AP shapes measured *in vitro* using current-clamp (grey), and recorded *in silico* (black). **B)** Quantification of the resting membrane potential (Vrmp), spike threshold potential, and the AP amplitude relative to the threshold, for 20 *in vitro* neurons and 20 *in silico* neurons where the first elicited AP was analysed. Data represent mean $\pm$ SEM. ns $P>0.05$, Mann-Whitney test was performed between two groups.

Supplemental experimental procedures

In silico sodium channel modification

To model the hypothesized changes in the sodium channel functioning in DS networks, we used a modification of the HH model that permits alteration of the activation, inactivation, conductance, and voltage sensitivity of the sodium channel. To model a shift to a persistent sodium current, we added a sodium current with infinitely fast activation m_∞ and slow inactivation h_p. The maximum persistent sodium conductance $\bar{g}_{\text{Nap}}$ was set to 0.1 mS $\cdot$ cm^{-2}. We incorporated the parameters γ_{Na}, γ_{Nap}, $\gamma_{\tau m}$, $\gamma_{\tau h}$, $\gamma_{\alpha m}$, $\gamma_{\alpha h}$, $\gamma_{\beta m}$, $\gamma_{\beta h}$, ΔV_m and ΔV_h into the HH equations:

$$\frac{dV_m}{dt} = \frac{1}{C_m}(-\bar{g}_{\text{K}} n^4 (V_m - E_{\text{K}}) - \boldsymbol{\gamma_{Na}}(1 - \boldsymbol{\gamma_{Nap}})\bar{g}_{\text{Na}} m^3 h (V_m - E_{Na}) - \bar{g}_l (V_m - E_l) \tag{1}$$

$$- \boldsymbol{\gamma_{Nap}}\bar{g}_{\text{Nap}} m_\infty h_p (V_m - E_{\text{Na}}) + I + I_{\text{sAHP}} + I_{\text{syn}}) + V_{\text{noise}},$$

$$\frac{dn}{dt} = \alpha_n (V_m)(1 - n) - \beta_n (V_m) n, \tag{2}$$

$$\frac{dm}{dt} = \boldsymbol{\gamma_{\tau m}}(\boldsymbol{\gamma_{\alpha m}}\alpha_m (V_m - \Delta V_m)(1 - m) - \boldsymbol{\gamma_{\beta m}}\beta_m (V_m - \Delta V_m) m, \tag{3}$$

$$\frac{dh}{dt} = \boldsymbol{\gamma_{\tau h}}(\boldsymbol{\gamma_{\alpha h}}\alpha_h (V_m - \Delta V_h)(1 - h) - \boldsymbol{\gamma_{\beta h}}\beta_h (V_m - \Delta V_h) h, \tag{4}$$

$$\frac{dh_p}{dt} = \alpha_h (V_m)(1 - h_p) - \frac{4}{1 + \exp[(V_m - V_T - 40)/5]} h_p, \tag{5}$$

where γ_{Na} modulates the maximum conductance of the sodium channels, which is analogous to altering the expression levels of the channel. $\gamma_{\tau m}$ and $\gamma_{\tau h}$ scale both rate constants α and β with the same factor so that effectively, the time constants, $\tau_i = \frac{1}{\alpha_i(V_m)+\beta_i(V_m)}$ of the $i = m$ and $i = h$ gate respectively, are scaled by $1/\gamma_{\tau i}$, while leaving the steady state, $i_\infty = \frac{\alpha_i(V_m)}{\alpha_i(V_m)+\beta_i(V_m)}$ unaffected. The kinetics of the sodium channel can also be modified by altering the rates of activation and deactivation of both the m and h gate individually using $\gamma_{\alpha m}$, $\gamma_{\alpha h}$, $\gamma_{\beta m}$ and $\gamma_{\beta h}$, leading to changes in both time constants and steady-states. The parameters ΔV_m and ΔV_h simultaneously shift the voltage sensitivity of both rate constants of the m and h gate, respectively. $\Delta V_i >$ 0 corresponds to a depolarizing shift in the voltage dependency and $\Delta V_i <$ 0 to a hyperpolarizing shift. An increase in γ_{Nap} models shifts the balance between the regular and persistent sodium current towards the persistent current. Note that γ_{Na} only increases maximal conductance of the regular sodium current. The control sodium channel model has parameter values $\gamma_{Na} = \gamma_{\tau m} = \gamma_{\tau h} = \gamma_{\alpha m} = \gamma_{\alpha h} = \gamma_{\beta m} = \gamma_{\beta h}$ = 1 and $\gamma_{Nap} = \Delta V_m = \Delta V_h$ = 0. We performed a parameter space exploration with these 10 parameters in order to map the possible effects of sodium channel modifications on the network dynamics.
