# Supplementary material for: An in silico and in vitro human neuronal network model reveals cellular mechanisms beyond NaV1.1 underlying Dravet syndrome
Source: Stem Cell Reports. 2023 Jul 6;18(8):1686–700. doi: 10.1016/j.stemcr.2023.06.003 (PMC10444571; doi:10.1016/j.stemcr.2023.06.003)
Supplement: Document S2. Article plus supplemental information [file mmc3.pdf]

Stem Cell Reports
Article
OPEN ACCESS

An *in silico* and *in vitro* human neuronal network model reveals cellular mechanisms beyond $Na_V1.1$ underlying Dravet syndrome

Nina Doorn,[1,7,*] Eline J.H. van Hugte,[2,3,4,7] Ummi Ciptasari,[3,4] Annika Mordelt,[3,4] Hil G.E. Meijer,[5] Dirk Schubert,[3,4] Monica Frega,[1] Nael Nadif Kasri,[3,4,8] and Michel J.A.M. van Putten[1,6,8]
[1]Department of Clinical Neurophysiology, University of Twente, 7522 NB Enschede, the Netherlands
[2]Department of Neurology, Academic Center for Epileptology Kempenhaeghe, 5591 VE Heeze, the Netherlands
[3]Department of Human Genetics, Radboudumc, 6500 HB Nijmegen, the Netherlands
[4]Department of Cognitive Neurosciences, Radboudumc, Donders Institute for Brain Cognition and Behaviour, 6525 HR Nijmegen, the Netherlands
[5]Department of Applied Mathematics, University of Twente, 7522 NB Enschede, the Netherlands
[6]Department of Neurology and Clinical Neurophysiology, Medisch Spectrum Twente, 7512 KZ Enschede, the Netherlands
[7]These authors contributed equally
[8]Senior author
*Correspondence: n.doorn-1@utwente.nl
https://doi.org/10.1016/j.stemcr.2023.06.003

SUMMARY

Human induced pluripotent stem cell (hiPSC)-derived neuronal networks on multi-electrode arrays (MEAs) provide a unique phenotyping tool to study neurological disorders. However, it is difficult to infer cellular mechanisms underlying these phenotypes. Computational modeling can utilize the rich dataset generated by MEAs, and advance understanding of disease mechanisms. However, existing models lack biophysical detail, or validation and calibration to relevant experimental data. We developed a biophysical *in silico* model that accurately simulates healthy neuronal networks on MEAs. To demonstrate the potential of our model, we studied neuronal networks derived from a Dravet syndrome (DS) patient with a missense mutation in *SCN1A*, encoding sodium channel $Na_V1.1$. Our *in silico* model revealed that sodium channel dysfunctions were insufficient to replicate the *in vitro* DS phenotype, and predicted decreased slow afterhyperpolarization and synaptic strengths. We verified these changes in DS patient-derived neurons, demonstrating the utility of our *in silico* model to predict disease mechanisms.

INTRODUCTION

Human induced pluripotent stem cell (hiPSC)-derived neuronal networks have become a key *in vitro* approach to study normal and abnormal formation of neural circuits, neurological disorders, or drug effects (Linda et al., 2022; Pires Monteiro et al., 2021; Yokoi et al., 2022). Networks cultured on multi-electrode arrays (MEAs) allow for non-invasive recording of neuronal network activity through embedded extracellular electrodes (Obien et al., 2015). *In vitro* neuronal networks derived from healthy subjects or patients show robust and replicable functional phenotypes (Mossink et al., 2021), and various genotype/phenotype correlations have been established (Frega et al., 2019; Marchetto et al., 2017; Klein Gunnewiek et al., 2021; Klein Gunnewiek et al., 2020). Despite these advances, the identification of cellular and synaptic mechanisms underlying the abnormal network phenotype remains challenging, as these are not trivial to deduce from the neuronal networks' electrical activity (Obien et al., 2015).

In silico modeling can complement experimental research and facilitate the identification of mechanisms underlying the observed neuronal network phenotype (Brodland 2015). Computational models have provided insight into various phenomena of *in vitro* neuronal networks such as network bursting (Masquelier and Deco 2013; Kumar et al., 2020), the effect of stimulation (Wen et al., 2022), and the role of astrocytes (Lenk et al., 2016). However, most of these *in silico* network models use phenomenological neuron models that can simulate simple neuronal behavior such as spikes, but do not describe how this behavior results from the intricate interplay of ion channels (Masquelier and Deco 2013; Wen et al., 2022; Lenk et al., 2016; Park et al., 2006; Pasquale et al., 2008; Mok et al., 2022). Moreover, most models are not calibrated and validated to data from human neuronal networks (Kumar et al., 2020; Trujillo et al., 2021; Wen et al., 2022; Masquelier and Deco 2013; Park et al., 2006; Lenk et al., 2016). Thus, the available models are ill-suited to study the effect of detailed cellular mechanisms on network activity and most lack the biophysical realism to simulate disorders that arise from channelopathies.

Here, we developed a biophysically detailed *in silico* model of hiPSC-derived excitatory neuronal networks on MEA, that accurately reproduces the main bursting activity measured in *in vitro* control neuronal networks. Furthermore, blocking of particular ion channels *in silico* and *in vitro* resulted in similar network phenotypes, serving as model validation. To illustrate the potential of the model to test hypotheses and predict factors underlying disease mechanisms, we investigated excitatory neuronal networks derived from a Dravet syndrome (DS) patient. DS is

 © 2023 The Author(s).
This is an open access article under the CC BY license (http://creativecommons.org/licenses/by/4.0/).

a severe infantile epileptic encephalopathy, caused by *de novo* mutations in *SCN1A* (Takayama et al., 2014; Dravet 2011; Ragona 2011), encoding the α-1 subunit of the voltage-gated sodium channel $Na_V1.1$ (Dravet 2011). It remains unresolved how excitatory neurons are affected by changes in sodium channel dynamics, and how this contributes to epileptogenesis (Isom 2014). Our *in silico* model revealed that sodium channel dysfunctions were insufficient to transition from a model of healthy networks to one that resembles the *in vitro* DS network behavior, and that additional alterations were needed. In particular, based on systematic model simulations, we predicted reduced slow afterhyperpolarization and synaptic strengths in the DS neuronal networks, which was subsequently confirmed *in vitro*. These results illustrate the utility of our *in silico* model to identify important mechanisms that can then be investigated *in vitro* in a targeted manner, expanding our understanding of disease mechanisms.

RESULTS

Our *in silico* model accurately reproduces activity of hiPSC-derived neuronal networks

To allow *in silico* investigation of cellular and network mechanisms of hiPSC-derived neuronal networks on MEA, we developed a biophysically detailed computational model (Figure 1B). The model consisted of 100 Hodgkin-Huxley (HH)-type neurons with voltage-gated sodium and potassium channels, leak channels accounting for the natural permeability of the neural membrane, and a slow, spike-dependent afterhyperpolarizing current (sAHP), corresponding to slow calcium- and sodium-activated potassium currents. The neurons were sparsely connected via synapses modeling AMPA receptors (AMPAr) and NMDA receptors (NMDAr), including short-term depression (STD). In this way, the model allows the investigation of the effect of specific changes to both ion channels and synaptic processes. The *in silico* model contained "virtual electrodes" similar to *in vitro* electrodes to represent the network activity. We based parameter choices on experimental measurements where possible, and chose the remaining parameters such that the simulated activity resembled the activity observed from *in vitro* control neuronal networks. The *in vitro* neuronal networks were differentiated from hiPSCs derived from a healthy subject through forced expression of *Ngn2* (Frega et al., 2017), and activity was recorded at 37 days *in vitro* (DIV) (Figure 1A). Both *in vitro* and *in silico* network activity consisted of spikes and bursts (short periods of high-frequency firing), which self-organized into synchronous network bursts (NBs) recorded in all electrodes (Figures 1C and 1D). *In vitro* neuronal networks exhibited stable periodic NBs, which were highly reproducible and previously observed across 10 independent control lines (Mossink et al., 2021). To quantitatively compare *in silico* simulations to *in vitro* measurements, we defined three NB features that remained stable over time (Mossink et al., 2021), the NB rate (NBR), the NB duration (NBD), and the percentage of spikes in NBs (PSIB). We analyzed the *in vitro* measurements and *in silico* simulations identically. There were no significant differences between the NBR and NBD *in vitro* and *in silico*, and the PSIB was similar (Figure 1E), showing that our *in silico* model can accurately simulate the main features of *in vitro* neuronal networks.

The *in silico* and *in vitro* neuronal networks show similar network behavior after pharmacological interventions with specific ion channel blockers

After establishing that the *in silico* model accurately simulated control neuronal network behavior, we aimed to further validate the model. We therefore performed interventions to both *in vitro* and *in silico* control networks and compared the effect without adjusting any other model parameters. First, we blocked KCNQ potassium channels *in vitro* using linopirdine (1.5 μM) (Schnee and Brown 1998; Noda et al., 1998; Guan et al., 2011). KCNQ potassium channels underlie part of the sAHP (Larsson 2013), and we therefore halved the conductance of the sAHP channels *in silico*. The addition of linopirdine resulted in a substantial increase in the NBD, both *in vitro* and *in silico*, while the NBR and PSIB were unaffected (Figure 2). Second, we inhibited sodium channels using tetrodotoxin (TTX) (1 μM) *in vitro* and blocked all sodium channels *in silico*. In both cases, the activity was completely abolished (data not shown). Third, we blocked NMDArs *in vitro*, using NMDAr antagonist MK-801 (1 μM), and *in silico* by setting the NMDA conductance to zero. Both resulted in a decreased NBD with minimal effect on the NBR and PSIB (Frega et al., 2019) (Figure 2). Finally, we inhibited AMPArs both *in vitro*, using AMPAr antagonists 2,3-dioxo-6-nitro-1,2,3,4-tetrahydrobenzo[f]quinoxaline7-sulfonamide (NBQX) (50 μM) and 1-naphthyl acetyl spermine trihydrochloride (NASPM) (10 μM), and *in silico* by abolishing the AMPA conductance. All bursting behavior vanished in both cultures and simulations (Figure 2). Although the variability of the effects of most blockers, reflected by a reduced SEM was noticeably smaller in simulations compared with experiments, there were no significant differences between the observations *in vitro* and *in silico* (Figure 2B). Because there were no remaining NBs after AMPA blockage, we also quantified the mean firing rate (MFR). There was a considerable reduction in MFR upon AMPA blockage, both *in vitro* and *in silico*. Nevertheless, the reduction ([mean ± SEM] 0.27 ± 0.08 spikes/s *in vitro* and 0.43 ± 0.04 spikes/s *in silico*) was significantly different ($p = 0.0163$) between the *in vitro* and *in silico* observations. Our observations, both *in vitro* and *in silico*, are in line with previous

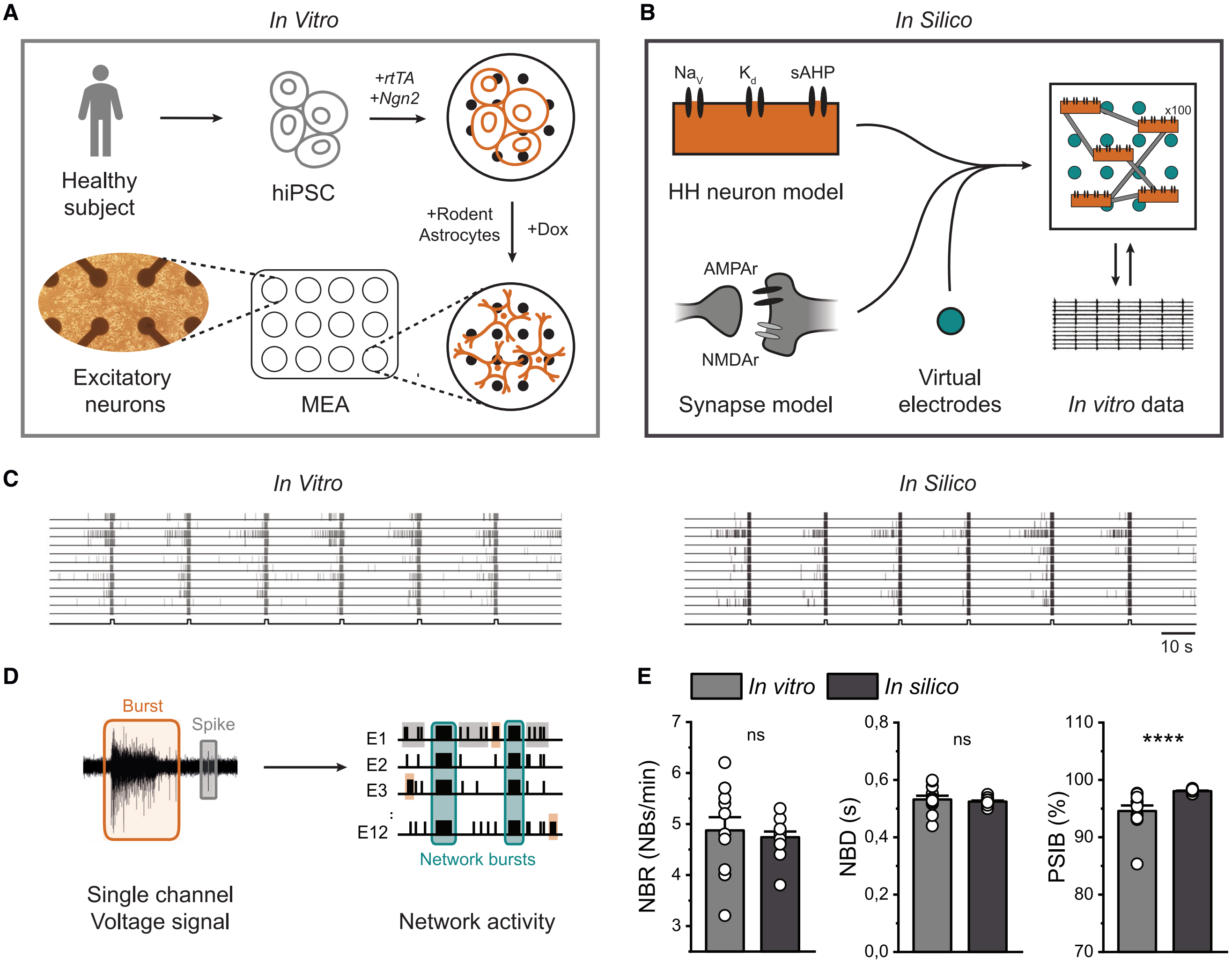

Figure 1. The *in silico* model resembles the network phenotype of hiPSC-derived excitatory neuronal networks
(A) Schematic overview of the differentiation protocol. hiPSCs were obtained by reprogramming somatic cells of a healthy subject. Excitatory neurons were generated through doxycycline (Dox)-inducible overexpression of Neurogenin2 (*Ngn2*). At 2 days *in vitro* (DIV), embryonic day 18 (E18) rodent astrocytes were added in a 1:1 ratio. Activity was recorded at 37 DIV.
(B) Schematic overview of the biophysical *in silico* model consisting of 100 Hodgkin-Huxley (HH)-type neurons, containing voltage-dependent sodium- (Na_V), delayed rectifier potassium (K_d), and slow afterhyperpolarizing currents (sAHPs), sparsely connected via AMPA receptor (AMPAr)- and NMDA receptor (NMDAr)-mediated synapse models, using virtual electrodes to measure network activity. The model is calibrated and validated to *in vitro* data.
(C) Representative raster plots showing 100 s of activity from a control network *in vitro* (left) and *in silico* (right). Black lines at the bottom of the raster plots indicate detected network bursts (NBs).
(D) Schematic overview of the analysis of MEA and "virtual electrode" signals.
(E) Quantification of network burst rate (NBR), network burst duration (NBD), and percentage of spikes in network bursts (PSIB) for 12 wells *in vitro* and 12 simulated networks *in silico*. Data represent mean ± SEM ns $p > 0.05$, ****$p < 0.0001$, Mann-Whitney test was performed between two groups.

literature describing the effect of ion channel blockers on neuronal network behavior (Frega et al., 2019; Kasteel and Westerink 2017). Thus, we conclude that our *in silico* model is an adequate computational representation of the *in vitro* recorded control excitatory neuronal networks.

The *in silico* model predicts alterations to the sAHP and synaptic strengths in DS neuronal networks

To assess the potential of the model to generate meaningful hypotheses about underlying disease mechanisms, we studied neuronal networks derived from a DS patient with

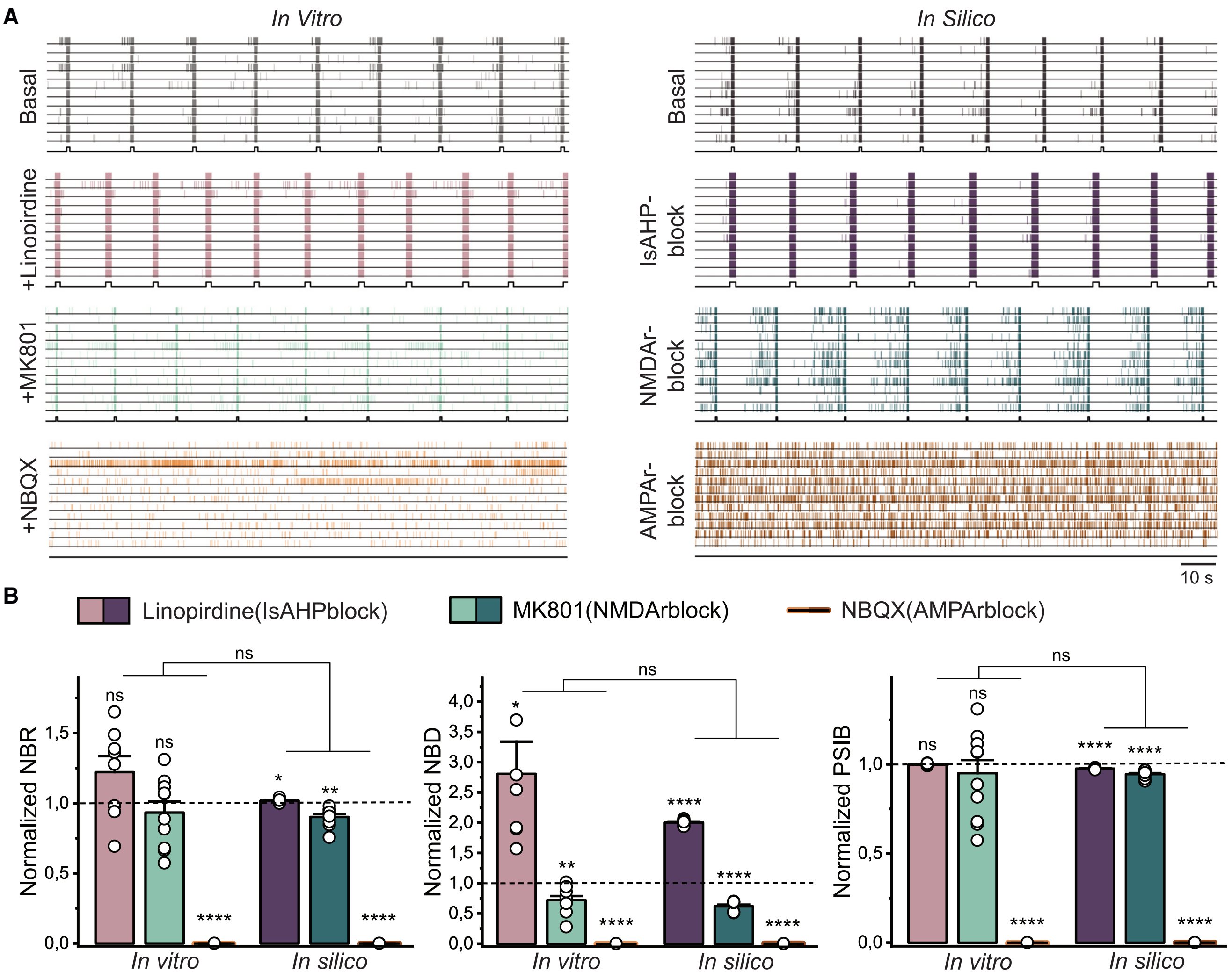

Figure 2. Blocking specific ion channels *in vitro* and *in silico* results in similar network phenotypes

(A) Left: representative raster plots showing 100 s of spontaneous activity from a control network *in vitro* in basal conditions, treated with 1.5 μM linopirdine to block KCNQ potassium currents, 1 μM MK-801 to block NMDArs, and 50 μM NBQX and 10 μM NASPM to block AMPArs. Right: representative raster plots showing 100 s of simulated activity from the *in silico* model in basal conditions, when the conductance of the sAHP channels is halved to model the effect of linopirdine, when all the NMDArs are blocked, and when all AMPArs are blocked. Black lines at the bottom of the raster plots indicate detected NBs.

(B) Quantification of the normalized NBD, normalized NBR, and normalized PSIB *in vitro* and *in silico* with 8 networks for linopirdine (sAHPblock), 10 networks for MK-801 (NMDArblock), and 4 networks for NBQX (AMPArblock) per condition. Data represent mean ± SEM ns $p > 0.05$, *$p < 0.05$, **$p < 0.005$, ****$p < 0.0001$. Groups were compared using a mixed effect model with multiple comparisons and Bonferroni correction.

a heterozygous missense mutation in the pore domain of *SCN1A* (c.4168G>A p.Val1390Met). DS neuronal networks showed less frequent NBs with a significantly longer duration compared with control (Figures 3C and 3D). Moreover, DS networks exhibited more spiking outside the NBs, resulting in a lower PSIB (Figure 3D). Thus, excitatory neuronal networks derived from DS patients show a distinct phenotype on MEA, which was in line with the addition of proconvulsive compounds in previous literature (Bradley et al., 2018). Previous modeling studies argued that alterations in sodium channel dynamics could explain the DS phenotypes (Berecki et al., 2019; Kahlig et al., 2006). To test this hypothesis, we explored *in silico* all sodium channel alterations that could arise from mutations in *SCN1A* to transition from the control neuronal network model to a DS model that resembled the situation *in vitro* (see supplemental experimental procedures and Figure S1A). We observed that every possible

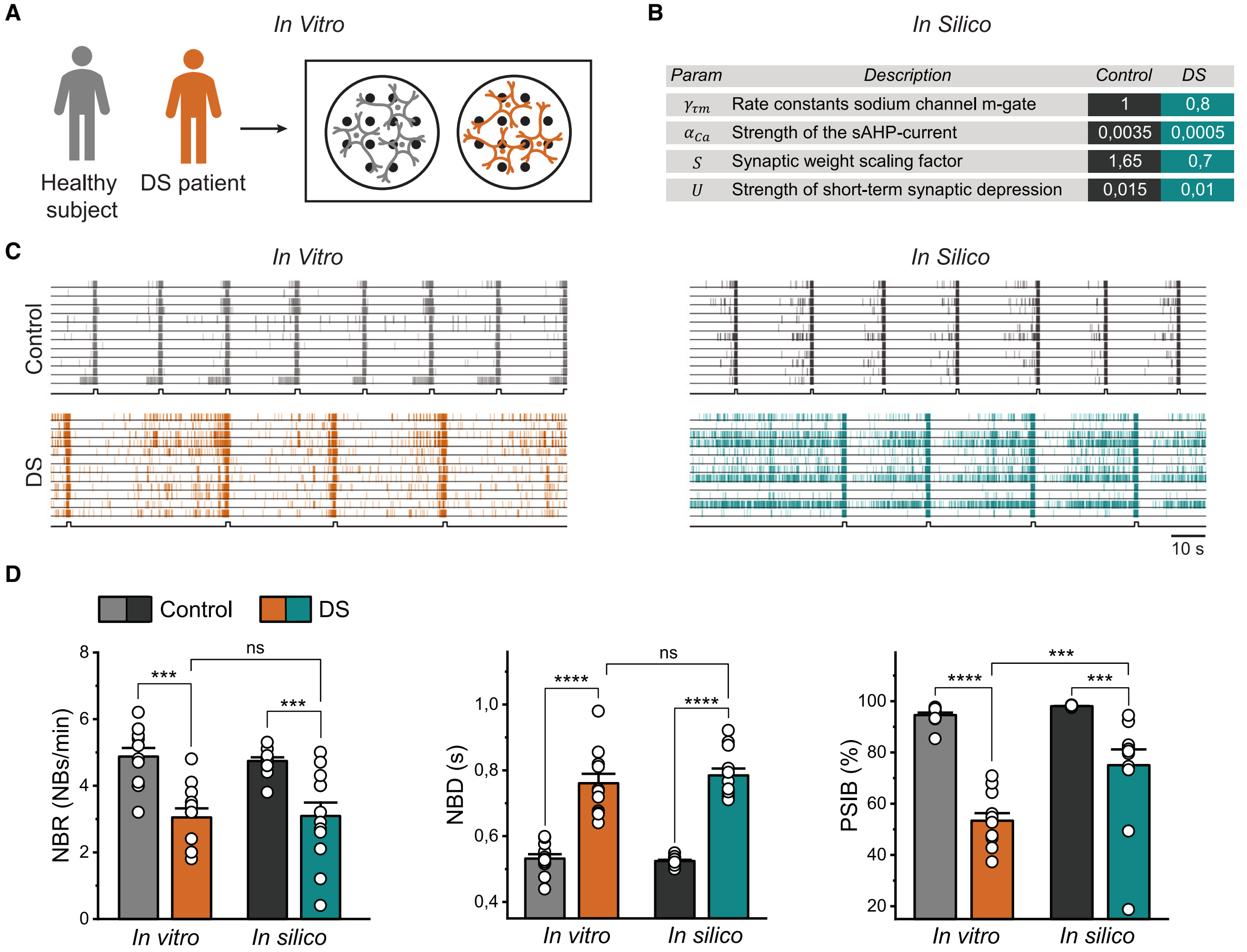

Figure 3. *In silico* model of DS neuronal networks replicates *in vitro* phenotype by altering sAHP and synaptic strengths

(A) Schematic overview of the *in vitro* data.

(B) Table with *in silico* altered parameters to transition from a model of control networks to a model of DS neuronal networks.

(C) Representative raster plots showing 100 s of spontaneous activity from an *in vitro* control network and an *in vitro* DS network (left), and simulated activity from the control and the DS *in silico* model (right). Black lines at the bottom of the raster plots indicate detected NBs.

(D) Quantification of NBR, NBD, and PSIB for 12 wells per condition *in vitro* and 12 simulated networks per model *in silico*. Data represent mean ± SEM, ns $p > 0.05$, ***$p < 0.0005$, ****$p < 0.0001$. Means were compared with a two-way ANOVA with Bonferroni correction for multiple testing.

(combination of) sodium channel modification(s) could qualitatively only either increase or decrease the neuronal excitability. On a network level, this led to either an increased or decreased NBR, while other features were largely unaffected (Figure S1C). In DS cultures, we observed a lower NBR compared with control, but also a lower PSIB and higher NBD (Figure S1B). In simulations with sodium channel modifications, the PSIB never significantly decreased and the NBD never increased in combination with a decreased NBR, comparable with the *in vitro* situation (Figure S1D). This suggests that changes in sodium channel dynamics are insufficient to transition from a functional phenotype of a control *in silico* model to a model of DS networks.

Since sodium channel modifications were insufficient, we explored other alterations, in particular synaptic properties and adaptive mechanisms. We observed that decreasing the conductance of the sAHP channels and decreasing the synaptic strengths (i.e., the magnitude of the excitatory post-synaptic currents [EPSCs]) lowered both the PSIB and NBR while increasing the NBD, similar to *in vitro* observations. Furthermore, we slightly reduced the amount of STD to obtain an NBD matching the *in vitro* observations. Although we could thus model the DS phenotype *in silico*

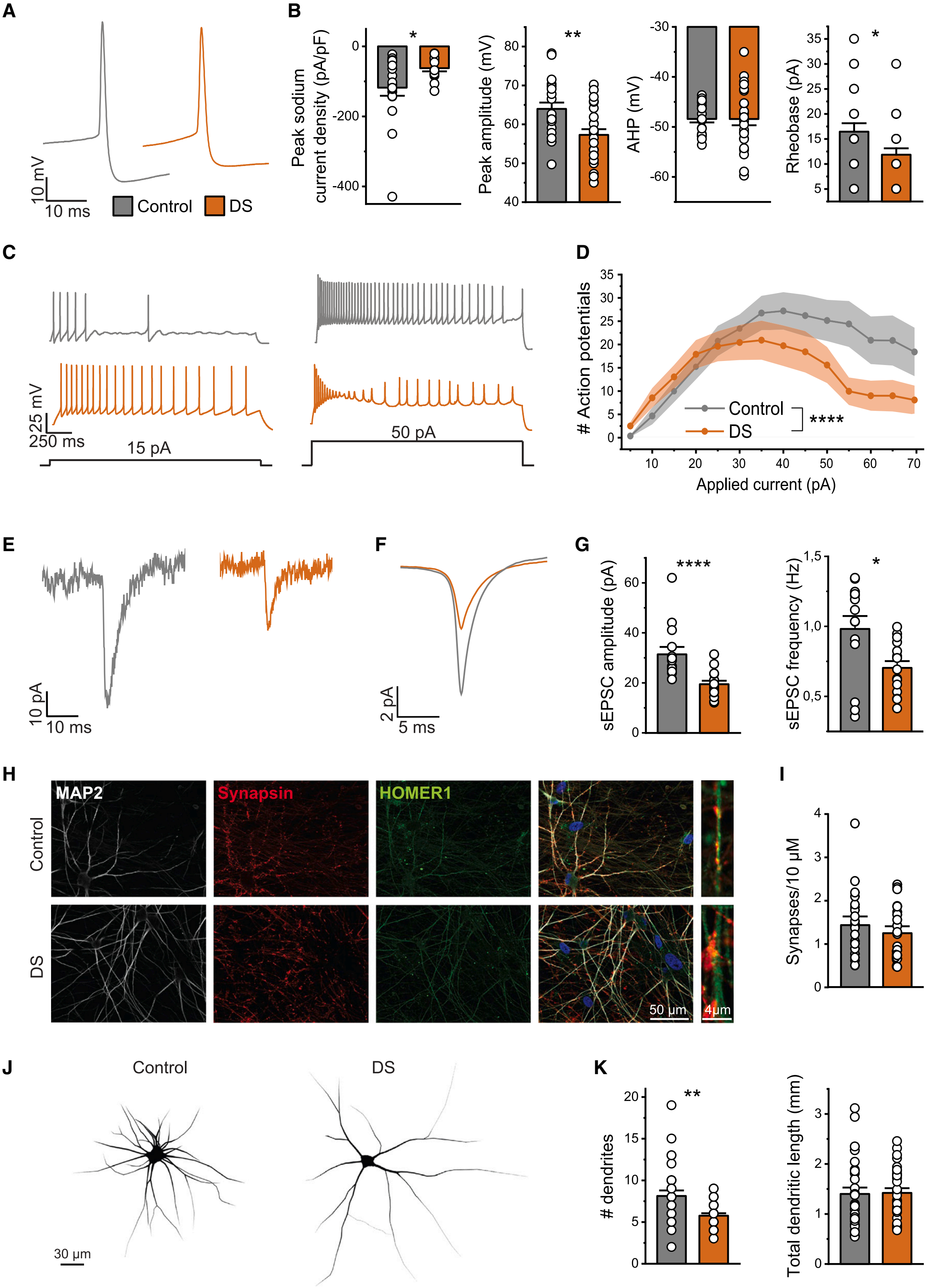

(legend on next page)

independent of sodium channel modifications, the DS neurons *in vitro* exhibited decreased peak sodium current. To mimic this, we decreased the rate constants of the sodium channel activation gate *in silico*. This did not change the *in silico* modeled DS network phenotype, confirming that the alterations in sAHP, synaptic strength, and STD could still reproduce a DS network phenotype in a DS biophysically relevant context. An overview of the modified parameters can be found in Figure 3B. The obtained model could replicate the differences between the *in vitro* observed control and DS neuronal network phenotypes (Figure 3C). There were no significant differences between the NBR and NBD of the *in vitro* and *in silico* DS models (Figure 3D). While there was a significant difference in PSIB between *in silico* and *in vitro* DS networks, the difference in PSIB between healthy and DS networks was similar *in vitro* and *in silico*. These results suggest that the DS excitatory neuronal network phenotype arises from disease mechanisms beyond $Na_V1.1$.

The *in silico* generated hypotheses are substantiated *in vitro*

The candidate cellular mechanisms in DS networks identified with our *in silico* model were subsequently investigated *in vitro*. We hypothesized that reduced sAHP, modeled as a potassium current that can be both calcium and sodium activated, could result from either reduced calcium or sodium currents. To this end, we measured the peak sodium current density and AP intrinsic properties using whole-cell current-clamp recordings. We observed decreased peak sodium current densities in DS neurons and a reduced AP amplitude, indicative of a reduction in both sodium- and voltage-dependent calcium currents (Gerlach et al., 2004). Similar to the *in silico* neurons, DS neurons *in vitro* were more excitable, reflected by a decreased rheobase, increased number of APs at lower current injections, and earlier depolarization block (Figures 4B–4D). To test the *in silico* prediction of reduced synaptic strengths in DS networks, we measured spontaneous EPSCs (sEPSCs) in control and DS network neurons using whole-cell voltage clamp. Indeed, both the sEPSC amplitudes and frequencies were significantly decreased in DS neurons (Figures 4E–4G). We found no significant difference in the synapse density or total dendritic length between control and DS neurons (Figures 4H and 4J), indicating that the decreased sEPSC amplitude and frequency are attributable to reduced synaptic strengths, and not to a decrease in the number of synapses. To conclude, DS neurons *in vitro* were hyperexcitable, and showed reductions in synaptic strengths, confirming the *in silico* predictions.

DISCUSSION

We here describe an *in silico* network model with detailed neuron dynamics, successfully calibrated and validated to experimental data from hiPSC-derived neuronal networks, able to generate meaningful hypotheses confirmed *in vitro*. Our model can be used to distinguish the effect of specific cellular changes on network dynamics, allowing for hypothesis testing and the development of mechanistic theories. With this model, researchers can leverage MEA data to identify potential cellular mechanisms underlying the neuronal network behavior, even if they lack the resources to do so *in vitro*.

Previously available models of *in vitro* neuronal networks were either not validated to data from human networks (Park et al., 2006; Masquelier and Deco 2013; Kumar et al., 2020; Pasquale et al., 2008; Trujillo et al., 2021), showed limited agreement between experimental and simulated data (Wen et al., 2022; Lenk et al., 2016), or used phenomenological neuron models, without detailed ion channel dynamics (Wen et al., 2022; Masquelier and Deco 2013; Park et al., 2006; Pasquale et al., 2008; Mok et al., 2022; Lenk et al., 2016). In contrast, we used a detailed neuron model with a biophysical description of how APs are generated by the interplay of ion channels,

Figure 4. *In vitro* DS neurons show hyperexcitability and reduced synaptic strengths
(A) Representative AP shapes in control (gray) and DS neurons (orange).
(B) Quantification of the peak sodium current density, AP peak amplitude, afterhyperpolarization (AHP) potential, and rheobase of control and DS neurons.
(C) Representative voltage traces from control and DS neurons in response to an applied current of 15 and 50 pA.
(D) The number of APs per current injection. Two-way ANOVA with Geisser-Greenhouse correction was performed to compare groups. For all intrinsic property data n = 20 for control and n = 26 for DS (3 independent experimental replicates).
(E and F) Representative (E) sEPSC traces and (F) average sEPSC traces of control and DS neurons.
(G) Quantification of sEPSC amplitudes and frequency in control (n = 14) and DS (n = 15) neurons (2 independent experimental replicates).
(H and I) Representative images of (H) control and DS neurons stained for MAP2, Synapsin1/2, and Homer1 at 35 DIV for the (I) quantification of the synaptic density in control (n = 21) and DS (n = 22) neurons (2 independent experimental replicates).
(J) Representative reconstructions of control (left) and DS (right) neurons.
(K) Quantification of the number of dendrites and the total dendritic length of control (n = 30) and DS (n = 30) neurons (3 independent experimental replicates).
Data represent mean ± SEM *p < 0.05, **p < 0.005, ****p < 0.0001, Mann-Whitney test was performed between two groups.

which allows for in-depth modeling of channelopathies, and a high agreement with *in-vitro*-generated data. However, increasing the model complexity also increases the number of parameters, which can potentially lead to a higher parameter degeneracy (Prinz et al., 2004; Achard and De Schutter 2006), meaning that multiple parameter combinations can lead to similar behavior. In that case, tuning model parameters is more difficult and predictions are more ambiguous. Conversely, it is important to keep in mind the simplifications in our model, such as the use of a one-compartmental neuron model that neglects the possible influence of neuronal morphology, or the lack of inhibitory neurons both *in vitro* and *in silico*, even though they might play a considerable role in the studied diseases (Kurbatova et al., 2016; Jansen et al., 2021; Lemaire et al., 2021; Chizhov et al., 2017). Depending on the research question, it might be relevant to reduce model complexity or, conversely, to increase it by the inclusion of inhibitory neurons or neuronal morphology.

Although our *in silico* model reproduced most electrophysiological signatures of *in vitro* neuronal networks, the PSIB in simulations was slightly higher than observed experimentally. This is likely due to the higher connectivity *in silico*, necessary to compensate for the lower number of neurons, and resulting in a higher synaptic input during an NB and consequently rapid firing. When we dramatically increased the number of neurons and proportionally decreased the connectivity, we observed a decrease in PSIB while the overall behavior of the network remained similar (Figure S2). This might also explain the slightly higher MFR after AMPA blockage *in silico*. Another slight disparity between experiment and simulation was the variability of the effect of ion channel blockers used for validation, likely due to the biological variability in cultures (Volpato and Webber 2020; Strube et al., 2017). For example, while the number of NMDArs and the effectiveness of MK-801 *in vitro* might differ per culture, the contribution of NMDA currents was always the same *in silico*. Thus, blocking the NMDArs *in silico* had quantitatively the same effect in all networks.

To illustrate the potential of our *in silico* model to test and generate hypotheses, we applied it to DS patient-derived excitatory networks. Previous work investigating DS has identified several sodium channel dysfunctions in excitatory neurons but it remains unexplained how this influences neuronal network behavior and epileptogenesis (Yu et al., 2006; Stein et al., 2019; Han et al., 2012; Liu et al., 2013; Jiao et al., 2013; Xie et al., 2020). In our *in silico* model, alterations to the sodium channel could not explain the aberrant network behavior observed *in vitro*. To replicate the DS phenotype *in silico*, we decreased the contribution of sAHP currents, synaptic strength, and the strength of STD, giving rise to the hypothesis that mechanisms beyond $Na_V1.1$ might contribute to the DS phenotype in excitatory neuronal networks.

Our *in silico* model predicted that DS excitatory neurons were hyperexcitable, caused by a reduced sAHP. Multiple potassium conductances constitute the AHP, which is comprised of a fast (f), medium (m), and sAHP component. While the fAHP and mAHP are mostly voltage dependent, the sAHP is largely voltage independent but can be both calcium and sodium activated (Gerlach et al., 2004; Wallen et al., 2007). We did not observe a reduction in absolute AHP potential in DS neurons, both *in silico* and *in vitro*. Indeed, the effect of sAHP on the AHP potential is negligible (Larsson 2013). As we were unable to dissect the sAHP potential from the general AHP potential, we attempted to quantify the possible contributors to the sAHP. We observed a decreased peak sodium current density, which could in turn reduce the contribution of sodium-dependent potassium currents. These potassium channels exhibit a large outward current under physiological conditions, activated by TTX-sensitive sodium currents, the major sodium current in our *in vitro* model (Budelli et al., 2009; Hage and Salkoff 2012). Therefore, we speculate that reduced sodium currents might lead to reduced sodium-activated potassium currents, decreasing the sAHP as predicted by our *in silico* model. Another large contributor to the sAHP is the slow calcium-activated potassium current (Larsson 2013). The decreased AP amplitude observed in DS neurons *in vitro* could lead to a reduced calcium influx, which in turn lowers the activation of calcium-activated potassium currents. Spratt et al. (2021) observed a similar reduction in AP amplitude in an *Scn2a* knockout mouse model, and correspondingly explained neocortical pyramidal cell hyperexcitability by attenuated hyperpolarizing potassium currents. Thus, reduced potassium currents might explain excitatory neuronal hyperexcitability, as a secondary effect to *SCN1A* deficiency in DS. Future work should confirm this by dissecting the contribution of different voltage-, calcium-, and sodium-dependent potassium currents in DS neurons.

Although we measured neuronal hyperexcitability both *in vitro* and *in silico*, the network activity appeared less synchronized, reflected by a lower NBR and PSIB. Our *in silico* model predicted that this resulted from reduced synaptic strengths, which might be a homeostatic response to the hyperexcitability observed both *in vitro* and *in silico*. Homeostatic synaptic downscaling is observed in *in vitro* networks when the neuronal activity is artificially elevated (Siddoway et al., 2014; Frega et al., 2019). The primary mechanism for synaptic downscaling is the remodeling of AMPArs in the post-synaptic membrane leading to lower EPSC amplitudes (Turrigiano et al., 1998). We observed decreased sEPSC amplitudes and frequencies in DS cultures compared with control, without a reduced synapse

number. Therefore, lowered sEPSC amplitudes and frequencies might be a result of reduced synaptic function in the *in vitro* DS networks. Homeostatic plasticity might also cause a reduction in STD, as our *in silico* model predicted (Deperrois and Graupner 2020; Almog et al., 2022). Based on these findings, we hypothesize that DS neuronal network activity is persistently increased due to neuronal hyperexcitability and that, consequently, synapses are downscaled as a form of homeostatic plasticity. Future studies should confirm the disease mechanisms proposed here for DS networks, as we used only one patient/control line at one developmental time point, to give a comprehensible example of how the computational model can be used. A possible obstacle in tuning our *in silico* model to other patient lines is the difficulty in finding the optimal model parameters. This could be overcome with the use of parameter optimization techniques that are widely available and tested on similar neuronal models (Sunnaker et al., 2013; Goncalves et al., 2020).

In sum, we developed a biophysically detailed *in silico* model that faithfully replicates *in vitro* observations from hiPSC-derived neuronal networks. Our model is a valuable tool complementary to *in vitro* measurements to strengthen the conclusions derived from these data.

EXPERIMENTAL PROCEDURES

Resource availability

Corresponding author

Further information and requests for resources and reagents should be directed to and will be fulfilled by the corresponding author Nina Doorn (n.doorn-1@utwente.nl).

Materials availability

All reagents, vectors, or cell lines used in this study are available from the lead contact upon request with a completed materials transfer agreement.

Data and code availability

The python code to run simulations *in silico*, together with the MATLAB code for data analysis is published on GitLab (https://gitlab.utwente.nl/m7706783/mea-model). All data are available from the lead contact upon request.

iPSC generation and neuronal differentiation

Experiments concerning patient-derived cells were carried out after informed consent and approval by the medical ethical committee of the Radboudumc, Nijmegen (2018-4525). The healthy donor was a 30-year-old male, as described previously and characterized (Frega et al., 2019; Mandegar et al., 2016). The DS patient was female and 4 years of age at the time of sampling. Direct target sequencing showed a missense mutation in the pore domain of *SCN1A* (c.4168G>A p.Val1390Met), which was confirmed in the hiPSC line (see Figure S2A). Peripheral mononuclear blood cells (PBMCs) were derived from a blood sample during scheduled routine diagnostic testing. hiPSC lines were obtained by episomal-vector-based reprogramming of PBMCs (Takahashi and Yamanaka 2006), and expressed pluripotency markers and displayed normal karyotype (see Figure S2B). To ensure sufficient quality, hiPSCs were passaged 2 times a week but not more than 16 times, and hiPSCs were checked for microplasm contamination every 8 passages. hiPSC cells were differentiated into excitatory cortical Layer 2/3 neurons through doxycycline-inducible overexpression of Neurogenin2 (*Ngn2*) as described previously (Frega et al., 2017; Mossink et al., 2021). Neurons were cultured in a density of 600 neurons/mm^2 and co-cultured with embryonic day 18 rodent astrocytes in a 1:1 ratio to support maturation.

MEA recording

Spontaneous network activity was recorded using the 24-well MEA system (Multi Channel Systems, Reutlingen, Germany). Each well consisted of 12 gold electrodes with a diameter of 30 μm, spaced 300 μm. The network activity was recorded at 37 DIV for 600 s with a 10 kHz sampling frequency in a recording chamber maintained at 37°C/95% O_2/5% CO_2. The experimental design adhered to previously published guidelines (Mossink et al., 2021), and included experiments with a minimum of 12 wells per hiPSC line across two independent batches. Recordings were analyzed using a custom MATLAB (The MathWorks, Natick, MA) script as described under data comparison and statistics.

Pharmacology

For pharmacological experiments on MEA we used the following compounds: linopirdine (1.5 μM in MQ, Sigma, no. 105431-72-9), TTX (1 μM in MQ, Tocris, no. 1069), NASPM (10 μM in MQ, Tocris, no. 2766), MK 801 maleate (MK-801), (1 μM in DMSO, Tocris no. 0924), and NBQX (50 μM in DMSO, no. 0373). The DMSO concentration in each experiment remained below 0.5% v/v.

Single-cell electrophysiology

For single-cell electrophysiology, cells were visualized using an Olympus BX51WI upright microscope (Olympus Life Science, Center Valley, PA) and a DAGE-MTI IR-1000E (DAGE-MTI, Michigan, IN) camera. Data were acquired through a Digidata 1440-A digitizer and a MultiClamp 700B amplifier (Molecular Devices, San Jose, CA). The data were sampled at 20 kHz and filtered using a low-pass 1 kHz filter. Filamented patch pipettes, with open tip resistance of 5–7 MΩ, were pulled from borosilicate glass (Science Products, Hofheim, Germany) with a PC-10 micropipette puller (Narishige, London, UK). Coverslips were placed at the recording chamber continuously perfused with artificial cerebrospinal fluid (ACSF) containing 124 mM NaCl, 1.25 mM NaH_2PO_4, 3 mM KCl, 26 mM $NaHCO_3$, 11 mM glucose, 2 mM $CaCl_2$, 1 mM $MgCl_2$ (adjusted to pH 7.4). The ACSF was maintained constant at 37°C/95% O_2/5% CO_2. Recordings were not analyzed if series resistance was above 20 MΩ, or the series to membrane resistance was lower than a 1:10 ratio. AP intrinsic properties were measured in current clamp using a potassium-based internal solution containing 130 mM K-gluconate, 5 mM KCl, 10 mM HEPES, 2.5 mM $MgCl_2$, 2 mM Na_2-ATP, 0.4 mM Na_3-ATP, 10 mM Na-phosphocreatine, 0.6 mM EGTA (adjusted to pH 7.25 and osmolarity 290 mOsmol). Active intrinsic

properties were measured using a stepwise current injection protocol ranging from −30 to +50 pA, and determined by analyzing the first action potential that was elicited by the protocol. All intrinsic properties were analyzed using Clampfit 10.7 (Molecular Devices). Both sEPSCs and sodium currents were measured in voltage clamp using a cesium-based solution containing 115 mM $CsMeSO_3$, 20 mM CsCl, 10 mM HEPES, 2.5 mM $MgCl_2$, 4 mM Na_2-ATP, 0.4 mM Na_3-ATP, 10 mM Na-phosphocreatine, 0.6 mM EGTA (adjusted to pH 7.2 and osmolarity 304 mOsmol), and recorded in ACSF at −60 mV. sEPSCs amplitude and frequency were quantified using an in-house MATLAB script using a threshold-based method. For sodium current measurements, P/8 leak subtraction was used and CNQX was present in the ACSF to block all synaptic activity during recording. Cells were measured using a stepwise protocol, cell membrane potential was increased from a holding potential of −90 mV in 10 mV increments to 60 mV for 100 ms. Peak currents recorded at the corresponding voltage step were determined using Clampfit 10.7, and divided by the cell capacitance.

Immunocytochemistry and neuronal morphology

Before immunostaining, cells were fixated with 4% PFA/4% sucrose for 15 min. Fixated coverslips were washed 3 times, and permeabilized with Triton X-100 (0.2%, Sigma-Aldrich, no. T8787) for 10 min, followed by 1 h of blocking with normal goat serum (NGS) (5%, Invitrogen, no. 10000C). Primary antibodies raised against Synapsin (1:500, Merck Milipore, no. AB1543P), MAP2 (1:1,000, Synaptic Systems, no. 188004), and Homer (1:500, Synaptic Systems, no. 160 011) were diluted in 1% NGS, and incubated overnight at 4°C. After 10 PBS washes, secondary antibodies diluted in 1% NGS were incubated for 1 h. Coverslips were washed 10 times with PBS, and stained with Hoechst (0.01%, Thermo Fisher Scientific, no. H3570) for 10 min, followed by 10 washes. Coverslips were mounted in DAKO mounting medium (Agilent, no. S3023) on microscope slides. Images were taken on a Zeiss Axio Imager Z2 at 63× magnification, and Fiji was used to quantify Synapsin/Homer double-positive puncta (Schindelin et al., 2012). MAP2-stained neurons were digitally reconstructed using Neurolucida 360 software (version 11, MBF-Bioscience, Williston, ND). The number of dendrites and dendritic length were quantified from reconstructed images.

Computational model

Our *in silico* model consists of several existing submodels. Neuron, synapse, and network parameter values are based on experiments and literature where possible, and the remaining parameters were chosen such that the simulations resembled the activity from control cultures.

Neuron model

We used an HH-type neuron model (Hodgkin and Huxley 1952), with expressions for the rate constants well suited for cortical pyramidal neurons (Traub and Miles 1991), and adapted these expressions to experimentally observed single-cell electrophysiology:

$$\alpha_m = \frac{-0.32(V_m - V_T - 13)}{\exp\left[\frac{-(V_m - V_T - 13)}{4}\right] - 1},$$

$$\beta_m = \frac{0.28(V_m - V_T - 40)}{\exp[(V - V_T - 40)/5] - 1},$$

$$\alpha_h = 0.128\exp\left[\frac{-(V_m - V_T - 17)}{18}\right],$$

$$\beta_h = \frac{4}{1+\exp\left[\frac{-(V_m - V_T - 40)}{5}\right]},$$

$$\alpha_n = \frac{-0.032(V_m - V_T - 15)}{\exp\left[\frac{-(V_m - V_T - 15)}{5}\right] - 1},$$

$$\beta_n = 0.5\exp\left[\frac{-(V_m - V_T - 10)}{40}\right].$$

V_m is the membrane potential of the neuron. V_T allows for adjusting the spike threshold to our experimental data. Maximal conductances were taken from Traub and Miles (1991). Nernst potentials and V_T were adapted such that the simulated neurons had the same average resting membrane potential, spike threshold potential, and AP amplitude as our *in vitro* neurons (Figure S4). All parameter values can be found in Table 1.

Adaptation model. We included spike-frequency adaptation in every neuron in the form of an additional sAHP current I_{sAHP}, given by:

$$I_{sAHP} = g_{AHP}(V_m - E_K).$$

The current is modeled as a spike-activated potassium current. When a neuron exhibits a spike at time t_0, we increase the conductance g_{AHP} with an amount α_{Ca}. The conductance then decays with time constant τ_{AHP}. The resulting equation for the conductance is:

$$\frac{dg_{AHP}}{dt} = -\frac{g_{AHP}}{\tau_{AHP}} + \alpha_{Ca}\delta(t - t_0),$$

where the delta function $\delta(t - t_0)$, is zero for $t \neq t_0$ and leads to a step response at t_0 of size 1 (multiplied by α_{Ca}). The current might correspond to slow sodium- or calcium-dependent potassium currents, but also other fatigue mechanisms. τ_{AHP} is the apparent recovery timescale of these mechanisms combined, which was chosen in the 2–8 s range found in the literature (Masquelier and Deco 2013; Gerlach et al., 2004). Parameters for the control model can be found in Table 1.

Noise and heterogeneity. We induced voltage fluctuations in every neuron, using:

$$V_{noise} = \sigma\sqrt{g_l C_m}\xi,$$

where ξ is Gaussian white noise with zero mean and σ the standard deviation of the resulting noise in the membrane potential. This noise can mimic synaptic or membrane noise.

We made the neurons heterogeneously excitable by drawing the amplitude of their constant applied current I from a uniform distribution between −9.5 and 9.5 pA.

Thus, the resulting neuron equations are:

Table 1. Overview of the parameters of the control *in silico* model

Parameter	Description	Value	Unit
C_m	membrane capacitance	1	$\mu F \cdot cm^{-2}$
$\overline{g_K}$	maximum delayed rectifier potassium conductance	5	$mS \cdot cm^{-2}$
$\overline{g_{Na}}$	maximum voltage-gated sodium conductance	50	$mS \cdot cm^{-2}$
$\overline{g_l}$	leak conductance	0.3	$mS \cdot cm^{-2}$
E_K	Nernst potential of potassium	−80	mV
E_{Na}	Nernst potential of sodium	70	mV
E_l	Nernst potential of the leak current	−39.2	mV
V_T	potential to adapt spike threshold	−30.4	mV
α_{Ca}	strength of sAHP	0.0035	nS
τ_{AHP}	recovery timescale of sAHP currents	6	s
σ	standard deviation of the noisy input	4.1	mV
$\overline{g_{AMPA}}$	maximal conductance of AMPA	0.2808	nS
$\overline{g_{NMDA}}$	maximal conductance of NMDA	0.0981	nS
E_{AMPA}	Nernst potential of AMPA	0	mV
E_{NMDA}	Nernst potential of NMDA	0	mV
α_{NMDA}	multiplicative constant of NMDA dynamics	0.5	kHz
τ_{AMPA}	decay time for AMPA synapses	2	ms
$\tau_{NMDA,decay}$	decay time for NMDA synapses	100	ms
$\tau_{NMDA,rise}$	rise time for NMDA synapses	2	ms

$$\frac{dV_m}{dt} = \frac{1}{C_m}\left(- \overline{g_K} n^4 (V_m - E_K) - \overline{g_{Na}} m^3 h (V_m - E_{Na}) - \overline{g_l}(V_m - E_l) + I + I_{sAHP}\right) + V_{noise},$$

$$\frac{dn}{dt} = \alpha_n(V_m)(1-n) - \beta_n(V_m)n,$$

$$\frac{dm}{dt} = \alpha_m(V_m)(1-m) - \beta_m(V_m)m,$$

$$\frac{dh}{dt} = \alpha_h(V_m)(1-h) - \beta_h(V_m)h.$$

Synapses and plasticity

EPSCs *in vitro* had both AMPA and NMDA components. We added a synaptic current I_{syn} to the HH equations:

$$I_{syn}(t) = I_{AMPA}(t) + I_{NMDA}(t).$$

AMPARs are modeled as an ohmic conductance g_{AMPA} multiplied with the difference between the membrane potential V_m of the post-synaptic neuron and the Nernst potential E_{AMPA} of the AMPA channels (Roth and van Rossum, 2013). NMDAr-mediated conductance also depends on the post-synaptic voltage, caused by blocking of the pore of the NMDAr by a magnesium ion. When the cell depolarizes, the magnesium block is lifted. The fraction of unblocked channels is fitted to:

$$u(V_m) = \frac{1}{1+e^{-aV_m}[Mg^{2+}]_o/b},$$

where V_m is the membrane potential of the post-synaptic neuron and $[Mg^{2+}]_o$ is the extracellular magnesium concentration, which we took to be 1 mM as in Jahr and Stevens (1990). We set a = 0.062 mV^{-1} and b = 3.57 mM (Jahr and Stevens 1990). We assumed that the magnesium block changes instantaneously with voltage and is independent of the gating of the channel. The synaptic conductances can be modeled as maximal conductances of all the AMPA and NMDA channels, $\overline{g_{AMPA}}$ and $\overline{g_{NMDA}}$, respectively, times the fraction of open channels. The fraction of open NMDA channels is the sum of the fractions of open channels per synapse with pre-synaptic neuron j, s_j^{NMDA} , multiplied with the synaptic weight from pre-synaptic neuron j to the post-synaptic neuron w_j. The equations for both synaptic currents are thus given by:

$$I_{AMPA} = \overline{g_{AMPA}}(V_m - E_{AMPA})\sum_{j=1}^{N_E} w_j s_j^{AMPA},$$

$$I_{NMDA} = \overline{g_{NMDA}} u(V_m)(V_m - E_{NMDA})\sum_{j=1}^{N_E} w_j s_j^{NMDA}.$$

The fraction of open channels is given by:

$$\frac{ds_j^{AMPA}}{dt} = -\frac{s_j^{AMPA}}{\tau_{AMPA}} + \sum_k \delta\left(t - t_j^k - \Delta\right),$$

$$\frac{ds_j^{NMDA}}{dt} = -\frac{s_j^{NMDA}}{\tau_{NMDA,decay}} + \alpha_{NMDA} x_j^{NMDA}\left(1 - s_j^{NMDA}\right),$$

$$\frac{dx_j^{NMDA}}{dt} = -\frac{x_j^{NMDA}}{\tau_{NMDA,rise}} + \sum_k \delta\left(t - t_j^k - \Delta\right).$$

Here, x_j^{NMDA} is an auxiliary gating variable for NMDA, and α_{NMDA} is a multiplicative constant. The fraction of open channels increases every time the pre-synaptic neuron spikes at time t_j^k. $\tau_{NMDA,rise}$ and $\tau_{NMDA,decay}$ are the rise and decay times for the NMDA synapses and τ_{AMPA} the decay time for AMPA synapses. These equations and the values for the time constants were taken from Masquelier and Deco (2013). We neglected the AMPA rise time because it is very short (Masquelier and Deco 2013). Δ is a conduction delay. All synaptic weights are multiplied with a synaptic scaling factor S. We took synaptic weights w_{ij} from a normal distribution with a mean of 1 and a standard deviation of 0.7. We set values below 0 to 0, and values above 2 to 2. This caused about 1 in 15 synapses to perish. Other parameter values can be found in Table 1.

STD. All synapses are modulated by STD. We used the phenomenological model proposed by Markram et al. (1998). The model is based on the concept of synaptic resources, of which only a fraction, x, is available. The synaptic weight w_j is multiplied with x_j, where x_j obeys:

$$\frac{dx_j}{dt} = \frac{1 - x_j}{\tau_D} - Ux_j \sum_k \delta\left(t - t_j^k - \Delta\right),$$

where τ_D is the time constant of STD, set to be 813 ms (Markram et al., 1998). U is the strength of STD, set to $U = 0.015$ (Markram et al., 1998).

Network properties

We constructed a sparsely connected network of N = 100 neurons. Neurons were randomly connected with a connection probability of 30%. Neurons were placed on a grid, allowing for the inclusion of distance-dependent conduction delays and virtual electrodes. Every virtual electrode measured the weighted sum of the membrane potential of the surrounding neurons, mimicking MEA measurements.

Simulations

Simulations were performed with the Brian2 simulator (Stimberg et al., 2019) in a Python 3.9 environment. Differential equations were integrated using either the exponential Euler or Euler forward method. To account for noise with these procedures, we kept the noise term constant over the timestep, where this noise term is as described in the noise and heterogeneity section, but ξ is replaced by a random number drawn from a normal distribution and divided by $\sqrt{dt}$. This way, we implemented the Euler-Maruyama scheme. To mimic the *in vitro* measurement time and sampling frequency, simulations were 650 s long, with a timestep of 0.1 ms, where the first 50 s were discarded as transient. We simulated 12 networks per condition with different connectivities, initial synaptic weights, and neuron heterogeneity, to mimic the 12 experimental wells. We validated the computational model by comparing the effects of pharmacological intervention *in vitro* and *in silico*. *In vitro*, we blocked potassium channels, sodium channels, AMPArs, and NMDArs using the inhibitors described under pharmacology. *In silico*, we simulated 650 s of basal network behavior in 30 networks. Then, we halved the conductance of the sAHP current in 8 of these networks, blocked all sodium channels in 8 networks, blocked NMDArs in 10 networks, and blocked AMPArs in four networks, and simulated for 650 s.

Data comparison and statistics

Data obtained from *in vitro* and *in silico* "MEA" recordings were handled identically. Signals were filtered between 100 and 3,500 Hz using a fifth-order Butterworth filter. We detected APs using an amplitude threshold-based method, where the threshold was 4 times the root mean-square of the electrode signal. NB detection started when the total spike rate remained above a threshold (1/4th of the maximum spike rate) for 60 ms, and stopped when the spike rate dropped below a second threshold (1/100th of the maximum spike rate). The NB was excluded if more than 80% of the spikes originated from a single electrode. We defined three features that were representative of network behavior and the differences between the control and DS network phenotypes, which were the NBR in NBs per minute, the NBD in seconds, and the PSIB.

We performed statistical analysis using GraphPad Prism 5 (GraphPad Software, San Diego, CA). We ensured normal distributions using a Kolmogorov-Smirnov test. When normality was not ensured, non-parametric testing using a Mann-Whitney test was performed. Equal variances of the to-be-compared groups were ensured using Levene's test. When comparing two means of distributions with unequal variances, a Welch's t test was performed. When equal variances and normality were ensured, a Student's t test was performed. For multiple comparisons, we used one-way ANOVA with Bonferroni correction. For repeated measures (model validation) we used a mixed-effect model with Geisser-Greenhouse correction for unequal variability of differences and Bonferroni test for multiple comparisons. To compare groups with measurements at multiple conditions, we used a two-way ANOVA with Geisser-Greenhouse correction. p values <0.05 were considered significant in all cases. All summary data, statistics and p values can be found in Table S1.

SUPPLEMENTAL INFORMATION

Supplemental information can be found online at https://doi.org/10.1016/j.stemcr.2023.06.003.

AUTHOR CONTRIBUTIONS

N.D. developed the *in silico* model and designed and performed *in silico* experiments. E.J.H.v.H., U.C., and A.M. designed and performed *in vitro* experiments. N.D., E.J.H.v.H., and U.C. performed data analyses. E.J.H.v.H., M.F., and N.N.K. contributed to development of the *in vitro* model. N.D. and E.J.H.v.H. wrote the manuscript with input from all authors. H.G.E.M. and D.S. provided

conceptualization and intellectual content. M.F., N.N.K., and M.J.A.M.v.P. conceived and supervised the project.

ACKNOWLEDGMENTS

This work was supported by the Netherlands Organisation for Health Research and Development ZonMw grant 91217055 (to N.N.K.), BRAINMODEL ZonMw PSIDER program 10250022110003 (to N.N.K. and M.F.), the European Joint Programme on Rare Diseases JTC2020-SCN1A-up! (to N.N.K.), and the Dutch epilepsiefonds WAR 18-02 (to N.N.K.). We thank Judith Verhoeven for providing patient material.

CONFLICT OF INTERESTS

The authors declare no competing interests.

Received: January 10, 2023
Revised: June 7, 2023
Accepted: June 7, 2023
Published: July 6, 2023

REFERENCES

Achard, P., and De Schutter, E. (2006). Complex parameter landscape for a complex neuron model. PLoS Comput. Biol. *2*, e94. https://doi.org/10.1371/JOURNAL.PCBI.0020094.

Almog, Y., Mavashov, A., Brusel, M., and Rubinstein, M. (2022). Functional investigation of a neuronal microcircuit in the CA1 area of the Hippocampus reveals synaptic dysfunction in dravet syndrome mice. Front. Mol. Neurosci. *15*, 97. https://doi.org/10.3389/FNMOL.2022.823640/BIBTEX.

Berecki, G., Bryson, A., Terhag, J., Maljevic, S., Gazina, E.V., Hill, S.L., and Petrou, S. (2019). SCN1A gain of function in early infantile encephalopathy. Ann. Neurol. *85*, 514–525. https://doi.org/10.1002/ana.25438.

Bradley, J.A., Luithardt, H.H., Metea, M.R., and Strock, C.J. (2018). In vitro screening for seizure liability using microelectrode array technology. Toxicol. Sci. *163*, 240–253. https://doi.org/10.1093/TOXSCI/KFY029.

Brodland, G.W. (2015). How computational models can help unlock biological systems. Semin. Cell Dev. Biol. *47–48*, 62–73. https://doi.org/10.1016/j.semcdb.2015.07.001.

Budelli, G., Hage, T.A., Wei, A., Rojas, P., Jong, Y.J.I., O'Malley, K., and Salkoff, L. (2009). Na+-activated K+ channels express a large delayed outward current in neurons during normal physiology. Nat. Neurosci. *12*, 745–750. https://doi.org/10.1038/nn.2313.

Chizhov, A.V., Amakhin, D.V., and Zaitsev, A.V. (2017). Computational model of interictal discharges triggered by interneurons. PLoS One *12*, e0185752. https://doi.org/10.1371/JOURNAL.PONE.0185752.

Deperrois, N., and Graupner, M. (2020). Short-term depression and long-term plasticity together tune sensitive range of synaptic plasticity. H. Cuntz, ed. *16*, e1008265. https://doi.org/10.1371/journal.pcbi.1008265.

Dravet, C. (2011). The core Dravet syndrome phenotype. Epilepsia *52*, 3–9. https://doi.org/10.1111/j.1528-1167.2011.02994.x.

Frega, M., Linda, K., Keller, J.M., Gümüş-Akay, G., Mossink, B., van Rhijn, J.R., Negwer, M., Klein Gunnewiek, T., Foreman, K., Kompier, N., et al. (2019). Neuronal network dysfunction in a model for Kleefstra syndrome mediated by enhanced NMDAR signaling. Nat. Commun. *10*, 1–15. https://doi.org/10.1038/s41467-019-12947-3.

Frega, M., van Gestel, S.H.C., Linda, K., Van Der Raadt, J., Keller, J., Van Rhijn, J.R., Schubert, D., Albers, C.A., and Nadif Kasri, N. (2017). Rapid neuronal differentiation of induced pluripotent stem cells for measuring network activity on micro-electrode arrays. J. Vis. Exp. *119*, 54900. https://doi.org/10.3791/54900.

Gerlach, A.C., Maylie, J., and Adelman, J.P. (2004). Activation kinetics of the slow afterhyperpolarization in hippocampal CA1 neurons. Pflügers Archiv *448*, 187–196. https://doi.org/10.1007/S00424-003-1237-2/FIGURES/8.

Goncalves, P.J., Lueckmann, J.M., Deistler, M., Nonnenmacher, M., Öcal, K., Bassetto, G., Chintaluri, C., Podlaski, W.F., Haddad, S.A., Vogels, T.P., et al. (2020). Training deep neural density estimators to identify mechanistic models of neural dynamics. Elife *9*, 1–46. https://doi.org/10.7554/ELIFE.56261.

Guan, D., Higgs, M.H., Horton, L.R., Spain, W.J., and Foehring, R.C. (2011). Contributions of Kv7-mediated potassium current to sub- and suprathreshold responses of rat layer II/III neocortical pyramidal neurons. J. Neurophysiol. *106*, 1722–1733. https://doi.org/10.1152/jn.00211.2011.

Hage, T.A., and Salkoff, L. (2012). Sodium-activated potassium channels are functionally coupled to persistent sodium currents. J. Neurosci. *32*, 2714–2721. https://doi.org/10.1523/JNEUROSCI.5088-11.2012.

Han, S., Tai, C., Westenbroek, R.E., Yu, F.H., Cheah, C.S., Potter, G.B., Rubenstein, J.L., Scheuer, T., De La Iglesia, H.O., and Catterall, W.A. (2012). Autistic-like behaviour in Scn1a+/- mice and rescue by enhanced GABA-mediated neurotransmission. Nature *489*, 385–390. https://doi.org/10.1038/nature11356.

Hodgkin, A.L., and Huxley, A.F. (1952). A quantitative description of membrane current and its application to conduction and excitation in nerve. J. Physiol. *117*, 500–544. https://doi.org/10.1113/jphysiol.1952.sp004764.

Isom, L.L. (2014). It was the interneuron with the parvalbumin in the hippocampus!" "No, it was the pyramidal cell with the glutamate in the cortex!" searching for clues to the mechanism of dravet syndrome - the plot thickens. Epilepsy Curr. *14*, 350–352. https://doi.org/10.5698/1535-7597-14.6.350.

Jahr, C.E., and Stevens, C.F. (1990). Voltage dependence of NMDA-activated macroscopic conductances predicted by single-channel kinetics. J. Neurosci. *10*, 3178–3182. https://doi.org/10.1523/jneurosci.10-09-03178.1990.

Jansen, N.A., Perez, C., Schenke, M., Van Beurden, A.W., Dehghani, A., Voskuyl, R.A., Thijs, R.D., Ullah, G., Van Den Maagdenberg, A.M., and Tolner, E.A. (2021). Impaired θ-γ coupling indicates inhibitory dysfunction and seizure risk in a dravet syndrome

mouse model. J. Neurosci. *41*, 524–537. https://doi.org/10.1523/JNEUROSCI.2132-20.2020.

Jiao, J., Yang, Y., Shi, Y., Chen, J., Gao, R., Fan, Y., Yao, H., Liao, W., Sun, X.F., and Gao, S. (2013). Modeling Dravet syndrome using induced pluripotent stem cells (iPSCs) and directly converted neurons. Hum. Mol. Genet. *22*, 4241–4252. https://doi.org/10.1093/hmg/ddt275.

Kahlig, K.M., Misra, S.N., and George, A.L. (2006). Impaired inactivation gate stabilization predicts increased persistent current for an epilepsy-associated SCN1A mutation. J. Neurosci. *26*, 10958–10966. https://doi.org/10.1523/JNEUROSCI.3378-06.2006.

Kasteel, E.E.J., and Westerink, R.H.S. (2017). Comparison of the acute inhibitory effects of Tetrodotoxin (TTX) in rat and human neuronal networks for risk assessment purposes. Toxicol. Lett. *270*, 12–16. https://doi.org/10.1016/J.TOXLET.2017.02.014.

Klein Gunnewiek, T., Van Hugte, E.J.H., Frega, M., Guardia, G.S., Foreman, K.B., Panneman, D., Mossink, B., Linda, K., Keller, J.M., Schubert, D., et al. (2020). Mitochondrial dysfunction impairs human neuronal development and reduces neuronal network activity and synchronicity. Cell Rep., 720227. https://doi.org/10.1101/720227.

Klein Gunnewiek, T.M., Verboven, A.H.A., Pelgrim, I., Hogeweg, M., Schoenmaker, C., Renkema, H., Beyrath, J., Smeitink, J., de Vries, B.B.A., Hoen, P.B.A.C.'t., et al. (2021). Sonlicromanol improves neuronal network dysfunction and transcriptome changes linked to m.3243AG heteroplasmy in iPSC-derived neurons. Stem Cell Rep. *16*, 2197–2212. https://doi.org/10.1016/J.STEMCR.2021.07.002.

Kumar, R., Huang, Y.-T., Chen, C.-C., Tzeng, S.-F., and Chan, C.-K. (2020). Astrocytic regulation of synchronous bursting in cortical cultures: from local to global. Cereb. Cortex Commun. *1*, tgaa053. https://doi.org/10.1093/TEXCOM/TGAA053.

Kurbatova, P., Wendling, F., Kaminska, A., Rosati, A., Nabbout, R., Guerrini, R., Dulac, O., Pons, G., Cornu, C., Nony, P., et al. (2016). Dynamic changes of depolarizing GABA in a computational model of epileptogenic brain: insight for Dravet syndrome. Exp. Neurol. *283*, 57–72. https://doi.org/10.1016/J.EXPNEUROL.2016.05.037.

Larsson, H.P. (2013). What determines the kinetics of the slow afterhyperpolarization(sAHP) in neurons? Biophys. J. *104*, 281–283. https://doi.org/10.1016/J.BPJ.2012.11.3832.

Lemaire, L., Desroches, M., Krupa, M., Pizzamiglio, L., Scalmani, P., and Mantegazza, M. (2021). Modeling NaV1.1/SCN1A sodium channel mutations in a microcircuit with realistic ion concentration dynamics suggests differential GABAergic mechanisms leading to hyperexcitability in epilepsy and hemiplegic migraine. PLoS Comput. Biol. *17*, e1009239. https://doi.org/10.1371/JOURNAL.PCBI.1009239.

Lenk, K., Priwitzer, B., Ylä-Outinen, L., Tietz, L.H., Narkilahti, S., and Hyttinen, J.A. (2016). Simulation of developing human neuronal cell networks. Biomed. Eng. Online *15*, 1–16. https://doi.org/10.1186/S12938-016-0226-6/FIGURES/4.

Linda, K., Lewerissa, E.I., Verboven, A.H.A., Gabriele, M., Frega, M., Klein Gunnewiek, T.M., Devilee, L., Ulferts, E., Hommersom, M., Oudakker, A., et al. (2022). Imbalanced autophagy causes synaptic deficits in a human model for neurodevelopmental disorders. Autophagy *18*, 423–442. https://doi.org/10.1080/15548627.2021.1936777.

Liu, Y., Lopez-Santiago, L.F., Yuan, Y., Jones, J.M., Zhang, H., O'Malley, H.A., Patino, G.A., O'Brien, J.E., Rusconi, R., Gupta, A., et al. (2013). Dravet syndrome patient-derived neurons suggest a novel epilepsy mechanism. Ann. Neurol. *74*, 128–139. https://doi.org/10.1002/ana.23897.

Mandegar, M.A., Huebsch, N., Frolov, E.B., Shin, E., Truong, A., Olvera, M.P., Chan, A.H., Miyaoka, Y., Holmes, K., Spencer, C.I., et al. (2016). CRISPR interference efficiently induces specific and reversible gene silencing in human iPSCs. Cell Stem Cell *18*, 541–553. https://doi.org/10.1016/j.stem.2016.01.022.

Marchetto, M.C., Belinson, H., Tian, Y., Freitas, B.C., Fu, C., Vadodaria, K., Beltrao-Braga, P., Trujillo, C.A., Mendes, A.P.D., Padmanabhan, K., et al. (2017). Altered proliferation and networks in neural cells derived from idiopathic autistic individuals. Mol. Psychiatr. *22*, 820–835. https://doi.org/10.1038/MP.2016.95.

Markram, H., Wang, Y., and Tsodyks, M. (1998). Differential signaling via the same axon of neocortical pyramidal neurons. Proc. Natl. Acad. Sci. USA *95*, 5323–5328. https://doi.org/10.1073/pnas.95.9.5323.

Masquelier, T., and Deco, G. (2013). Network bursting dynamics in excitatory cortical neuron cultures results from the combination of different adaptive mechanism. PLoS One *8*, e75824. https://doi.org/10.1371/journal.pone.0075824.

Mok, R.S.F., Zhang, W., Sheikh, T.I., Pradeepan, K., Fernandes, I.R., DeJong, L.C., Benigno, G., Hildebrandt, M.R., Mufteev, M., Rodrigues, D.C., et al. (2022). Wide spectrum of neuronal and network phenotypes in human stem cell-derived excitatory neurons with Rett syndrome-associated MECP2 mutations. Transl. Psychiatry *12*, 450. https://doi.org/10.1038/s41398-022-02216-1.

Mossink, B., Verboven, A.H.A., van Hugte, E.J.H., Klein Gunnewiek, T.M., Parodi, G., Linda, K., Schoenmaker, C., Kleefstra, T., Kozicz, T., van Bokhoven, H., et al. (2021). Human neuronal networks on micro-electrode arrays are a highly robust tool to study disease-specific genotype-phenotype correlations in vitro. Stem Cell Rep. *16*, 2182–2196. https://doi.org/10.1016/J.STEMCR.2021.07.001.

Noda, M., Obana, M., and Akaike, N. (1998). Inhibition of M-type K+ current by linopirdine, a neurotransmitter-release enhancer, in NG108-15 neuronal cells and rat cerebral neurons in culture. Brain Res. *794*, 274–280. https://doi.org/10.1016/S0006-8993(98)00235-2.

Obien, M.E.J., Deligkaris, K., Bullmann, T., Bakkum, D.J., and Frey, U. (2015). Revealing neuronal function through microelectrode array recordings. Front. Neurosci. *9*, 423. https://doi.org/10.3389/fnins.2014.00423.

Park, I., Xu, D., Demarse, T.B., and Principe, J.C. (2006). Modeling of synchronized burst in dissociated cortical tissue: an exploration of parameter space. In IEEE International Conference on Neural Networks - Conference Proceedings, pp. 581–586. https://doi.org/10.1109/ijcnn.2006.246734.

Pasquale, V., Massobrio, P., Bologna, L.L., Chiappalone, M., and Martinoia, S. (2008). Selforganization and neuronal avalanches in networks of dissociated cortical neurons. Neuroscience *153*, 1354–1369. https://doi.org/10.1016/j.neuroscience.2008.03.050.

Pires Monteiro, S., Voogd, E., Muzzi, L., De Vecchis, G., Mossink, B., Levers, M., Hassink, G., Van Putten, M., Le Feber, J., Hofmeijer, J., and Frega, M. (2021). Neuroprotective effect of hypoxic preconditioning and neuronal activation in a in vitro human model of the ischemic penumbra. J. Neural. Eng. *18*, 036016. https://doi.org/10.1088/1741-2552/ABE68A.

Prinz, A.A., Bucher, D., and Marder, E. (2004). Similar network activity from disparate circuit parameters. Nat. Neurosci. *7*, 1345–1352. https://doi.org/10.1038/nn1352.

Ragona, F. (2011). Cognitive development in children with Dravet syndrome. Epilepsia *52*, 39–43. https://doi.org/10.1111/J.1528-1167.2011.03000.X.

Roth, A., and van Rossum, M.C.W. (2013). Modeling synapses. In Computational Modeling Methods for Neuroscientists (The MIT Press), pp. 139–160. https://doi.org/10.7551/mitpress/9780262013277.003.0007.

Schindelin, J., Arganda-Carreras, I., Frise, E., Kaynig, V., Longair, M., Pietzsch, T., Preibisch, S., Rueden, C., Saalfeld, S., Schmid, B., et al. (2012). Fiji: an open-source platform for biological-image analysis. Nat. Methods *9*, 676–682. https://doi.org/10.1038/nmeth.2019.

Schnee, M.E., and Brown, B.S. (1998). Selectivity of linopirdine (DuP 996), a neurotransmitter release enhancer, in blocking voltage-dependent and calcium-activated potassium currents in hippocampal neurons. J. Pharmacol. Exp. Therapeut. *286*, 709–717.

Siddoway, B., Hou, H., and Xia, H. (2014). Molecular mechanisms of homeostatic synaptic downscaling. Neuropharmacology *78*, 38–44. https://doi.org/10.1016/j.neuropharm.2013.07.009.

Spratt, P.W.E., Alexander, R.P.D., Ben-Shalom, R., Sahagun, A., Kyoung, H., Keeshen, C.M., Sanders, S.J., and Bender, K.J. (2021). Paradoxical hyperexcitability from NaV1.2 sodium channel loss in neocortical pyramidal cells. Cell Rep. *36*, 109483. https://doi.org/10.1016/j.celrep.2021.109483.

Stein, R.E., Kaplan, J.S., Li, J., and Catterall, W.A. (2019). Hippocampal deletion of NaV1.1 channels in mice causes thermal seizures and cognitive deficit characteristic of Dravet Syndrome. Proc. Natl. Acad. Sci. USA *116*, 16571–16576. https://doi.org/10.1073/PNAS.1906833116.

Stimberg, M., Brette, R., and Goodman, D.F. (2019). Brian 2, an intuitive and efficient neural simulator. Elife *8*, e47314. https://doi.org/10.7554/eLife.47314.

Strube, C., Gackière, F., Saliba, L., Tell, F., and Kessler, J.-P. (2017). Variability of quantal NMDA to AMPA current ratio in nucleus tractus solitarii neurons. bioRxiv. https://doi.org/10.1101/110569.

Sunnaker, M., Busetto, A.G., Numminen, E., Corander, J., Foll, M., and Dessimoz, C. (2013). Approximate bayesian computation. PLoS Comput. Biol. *9*, e1002803. https://doi.org/10.1371/JOURNAL.PCBI.1002803.

Takahashi, K., and Yamanaka, S. (2006). Induction of pluripotent stem cells from mouse embryonic and adult fibroblast cultures by defined factors. Cell *126*, 663–676. https://doi.org/10.1016/j.cell.2006.07.024.

Takayama, R., Fujiwara, T., Shigematsu, H., Imai, K., Takahashi, Y., Yamakawa, K., and Inoue, Y. (2014). Long-term course of Dravet syndrome: a study from an epilepsy center in Japan. Epilepsia *55.4*, 528–538. https://doi.org/10.1111/EPI.12532.

Traub, R.D., and Miles, R. (1991). Neuronal Networks of the Hippocampus (Cambridge University Press). https://doi.org/10.1017/cbo9780511895401.

Trujillo, C.A., Adams, J.W., Negraes, P.D., Carromeu, C., Tejwani, L., Acab, A., Tsuda, B., Thomas, C.A., Sodhi, N., Fichter, K.M., et al. (2021). Pharmacological reversal of synaptic and network pathology in human MECP2 -KO neurons and cortical organoids. EMBO Mol. Med. *13*, 1. https://doi.org/10.15252/EMMM.202012523.

Turrigiano, G.G., Leslie, K.R., Desai, N.S., Rutherford, L.C., and Nelson, S.B. (1998). Activity-dependent scaling of quantal amplitude in neocortical neurons. Nature *391*, 892–896. https://doi.org/10.1038/36103.

Volpato, V., and Webber, C. (2020). Addressing variability in iPSC-derived models of human disease: guidelines to promote reproducibility. DMM Disease Models and Mechanisms *13*. https://doi.org/10.1242/dmm.042317.

Wallén, P., Robertson, B., Cangiano, L., Löw, P., Bhattacharjee, A., Kaczmarek, L.K., and Grillner, S. (2007). Sodium-dependent potassium channels of a Slack-like subtype contribute to the slow afterhyperpolarization in lamprey spinal neurons. J. Physiol. *585*, 75–90. https://doi.org/10.1113/JPHYSIOL.2007.138156.

Wen, J., Peitz, M., and Brüstle, O. (2022). A defined human-specific platform for modeling neuronal network stimulation in vitro and in silico. J. Neurosci. Methods *373*, 109562. https://doi.org/10.1016/J.JNEUMETH.2022.109562.

Xie, Y., Ng, N.N., Safrina, O.S., Ramos, C.M., Ess, K.C., Schwartz, P.H., Smith, M.A., and O'Dowd, D.K. (2020). Comparisons of dual isogenic human iPSC pairs identify functional alterations directly caused by an epilepsy associated SCN1A mutation. Neurobiol. Dis. *134*, 104627. https://doi.org/10.1016/J.NBD.2019.104627.

Yokoi, R., Shigemoto-Kuroda, T., Matsuda, N., Odawara, A., and Suzuki, I. (2022). Electrophysiological responses to seizurogenic compounds dependent on E/I balance in human iPSCderived cortical neural networks. J. Pharmacol. Sci. *148*, 267–278. https://doi.org/10.1016/J.JPHS.2021.12.006.

Yu, F.H., Mantegazza, M., Westenbroek, R.E., Robbins, C.A., Kalume, F., Burton, K.A., Spain, W.J., McKnight, G.S., Scheuer, T., and Catterall, W.A. (2006). Reduced sodium current in GABAergic interneurons in a mouse model of severe myoclonic epilepsy in infancy. Nat. Neurosci. *9*, 1142–1149. https://doi.org/10.1038/NN1754.

Stem Cell Reports, Volume *18*

Supplemental Information

An *in silico* and *in vitro* human neuronal network model reveals cellular mechanisms beyond $Na_V1.1$ underlying Dravet syndrome

Nina Doorn, Eline J.H. van Hugte, Ummi Ciptasari, Annika Mordelt, Hil G.E. Meijer, Dirk Schubert, Monica Frega, Nael Nadif Kasri, and Michel J.A.M. van Putten

Supplemental Figures

A

Parameter	*Modulated part of NaV1.1*	*Effect of increasing value*
γ_{Na}	Maximal Conductance	Hyperactive neurons
$\gamma_{\tau m}$	Both rate constants m-gate	Hyperactive neurons
$\gamma_{\tau h}$	Both rate constants h-gate	Hyperactive neurons
$\gamma_{\alpha m}$	Activation rate constant m-gate	Hyperactive neurons
$\gamma_{\beta m}$	Deactivation rate constant m-gate	Hypoactive neurons
$\gamma_{\alpha h}$	Activation rate constant h-gate	Hyperactive neurons
$\gamma_{\beta h}$	Deactivation rate constant h-gate	No effect
ΔV_m	Shift voltage sensitivity m-gate	Hypoactive neurons
ΔV_h	Shift voltage sensitivity h-gate	Hyperactive neurons
γ_{NaP}	Fraction of persistent sodium channels	Hyperactive neurons

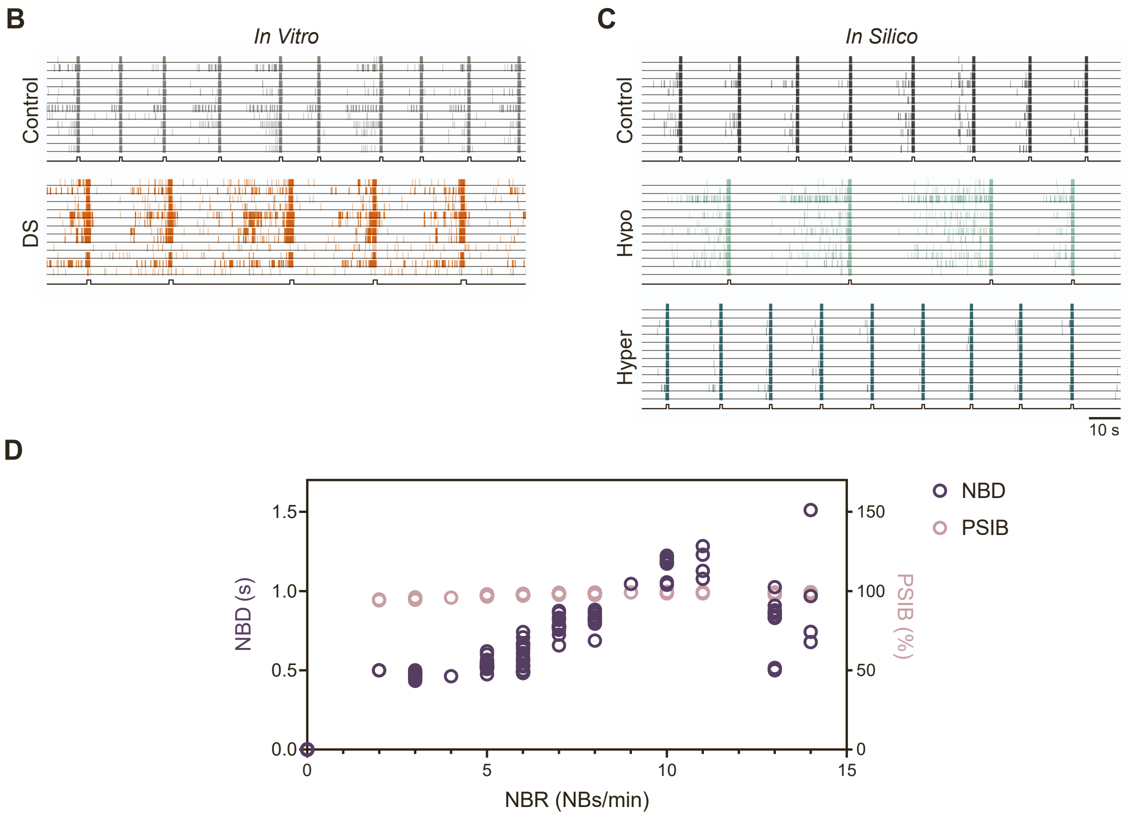

Figure S1: The effect of sodium channel modifications on the *in silico* neuron and network activity.
A) The effect of increasing the different modification parameter values (supplemental equations 1-5) on the excitability of the neuron. **B)** Representative raster plots showing 100 seconds of spontaneous activity of control and DS networks *in vitro*, with black lines indicating detected NBs. **C)** Representative raster plots showing simulations with the control *in silico* model as it is and when sodium channel modifications are made that result in hypoexcitable neurons (hypo) and hyperexcitable neurons (hyper). **D)** Quantification of the Network Burst Rate (NBR), Network Burst Dutation (NBD), and Percentage of Spikes in Network Bursts (PSIB) of the parameter space exploration with sodium channel modifications. NBD and PSIB are reported against the NBR from the corresponding simulations. The NBD in control never exceeded the NBD in DS networks with low NBR values, and the PSIB never decreased.

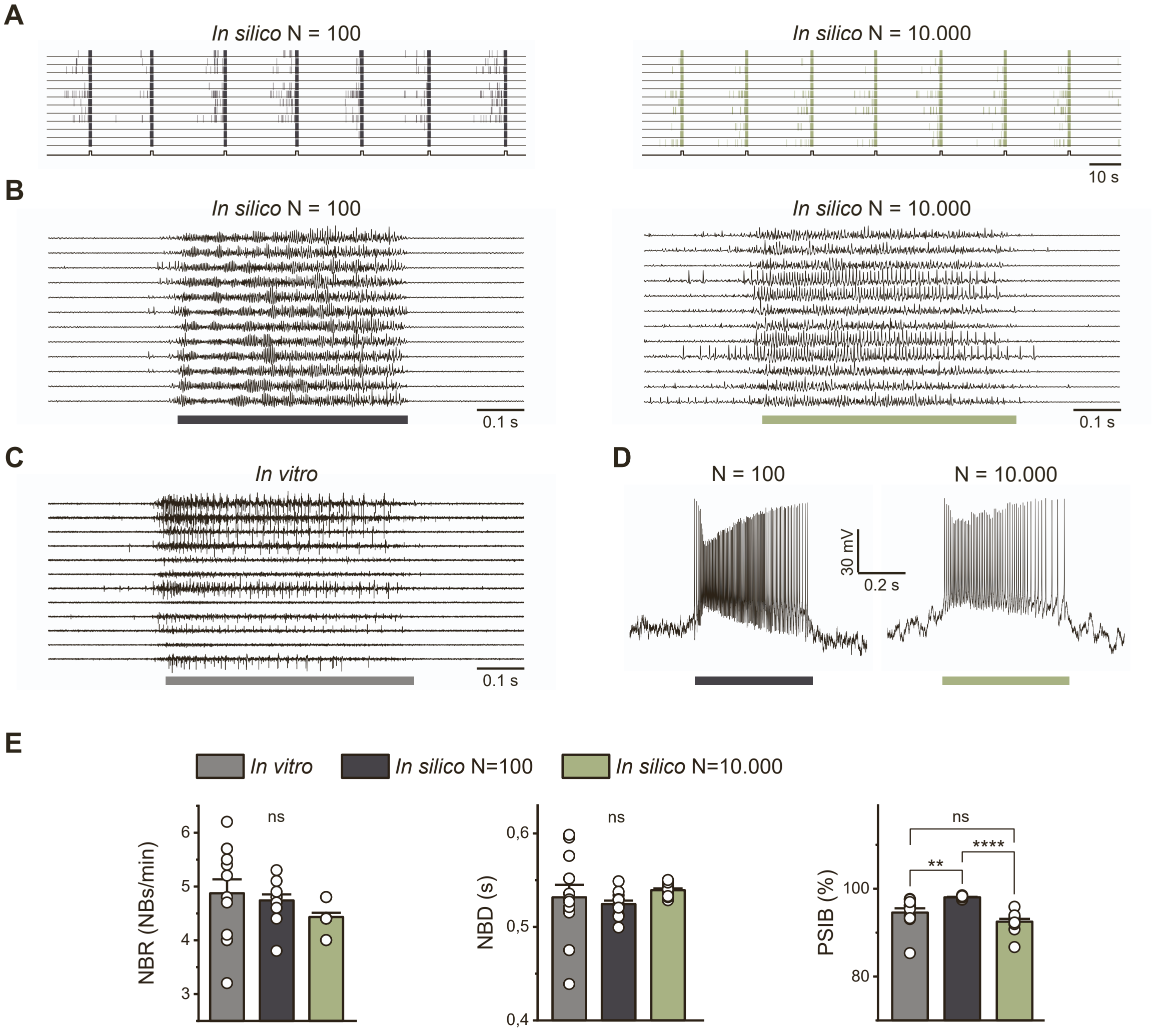

Figure S2: *In silico* simulations with 10.000 neurons result in a PSIB more comparable to control *in vitro* observations

A) Representative raster plots showing 100 seconds of simulated activity from the control *in silico* model with 100 neurons (left) and with 10.000 neurons (right). **B)** Voltage recordings from the virtual MEA electrodes showing an NB (marked below) in a simulation with 100 neurons (left) and with 10.000 neurons (right). **C)** Voltage recording from MEA electrodes showing an NB in an *in vitro* control network. **D)** The membrane potential of single neurons during an NB in a network simulation with 100 neurons (left) and 10.000 neurons (right). Due to the high synaptic input, neurons in the 100-neuron network go into depolarization block, resulting in MEA recordings with low-amplitude voltage fluctuations at the start of an NB. **E)** Quantification of Network Burst Rate (NBR), Network Burst Duration (NBD), and Percentage of Spikes in Network Bursts (PSIB) for 12 wells *in vitro* (600 s recordings) and 12 simulated networks per model *in silico* (600 s simulations with N=100 networks, and 150 s simulations with N=10.000 networks). Data represent mean $\pm$ SEM, ns $P>0.05$, ** $P<0.005$, **** $P<0.0001$. Means were compared with a two-way ANOVA with Bonferroni correction for multiple testing.

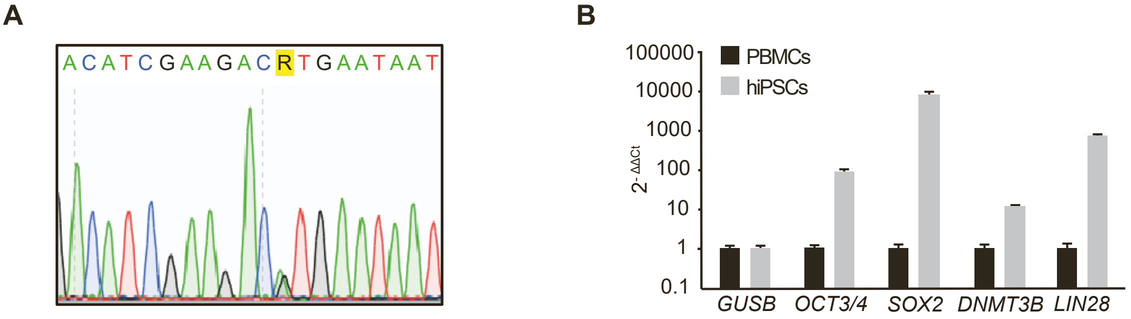

Figure S3: Mutation verification and pluripotency quantification.
A) Chromatogram of sequencing results depicting the heterozygous missense mutation c.4168G>A p.Val1390Met in *SCN1A* B) Quantitative real time PCR of pluripotentcy markers in hiPSCs relative to PBMC (perepheral mononuclear blood cells). Delta ct levels of octamer-binding transcription factor 3/4 (*OCT3/4*), SRY-box 2 (*SOX2*), *DNMT3B*, and *LIN28*, using glucuronidase beta (*GUSB*) as housekeeping gene, displayed as the relative gene expression normalized to *GUSB* levels.

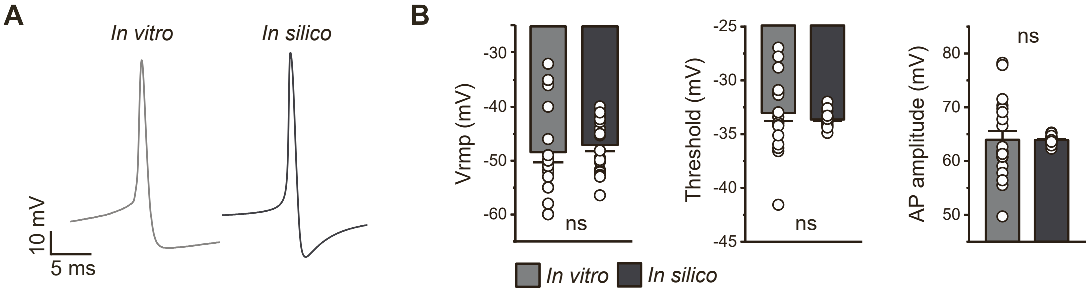

Figure S4: Comparison of *in vitro* and *in silico* intracellular AP waveshapes.
A) Representative AP shapes measured *in vitro* using current-clamp (grey), and recorded *in silico* (black). **B)** Quantification of the resting membrane potential (Vrmp), spike threshold potential, and the AP amplitude relative to the threshold, for 20 *in vitro* neurons and 20 *in silico* neurons where the first elicited AP was analysed. Data represent mean $\pm$ SEM. ns P>0.05, Mann-Whitney test was performed between two groups.

Supplemental experimental procedures

In silico sodium channel modification

To model the hypothesized changes in the sodium channel functioning in DS networks, we used a modification of the HH model that permits alteration of the activation, inactivation, conductance, and voltage sensitivity of the sodium channel. To model a shift to a persistent sodium current, we added a sodium current with infinitely fast activation m_∞ and slow inactivation h_p. The maximum persistent sodium conductance $\bar{g}_{\text{Nap}}$ was set to 0.1 mS $\cdot$ cm^{-2}. We incorporated the parameters γ_{Na}, γ_{Nap}, $\gamma_{\tau m}$, $\gamma_{\tau h}$, $\gamma_{\alpha m}$, $\gamma_{\alpha h}$, $\gamma_{\beta m}$, $\gamma_{\beta h}$, ΔV_m and ΔV_h into the HH equations:

$$\frac{dV_m}{dt} = \frac{1}{C_m}(-\bar{g}_{\text{K}} n^4 (V_m - E_{\text{K}}) - \boldsymbol{\gamma_{Na}}(1 - \boldsymbol{\gamma_{Nap}})\bar{g}_{\text{Na}} m^3 h (V_m - E_{Na}) - \bar{g}_l (V_m - E_l) \tag{1}$$

$$- \boldsymbol{\gamma_{Nap}}\bar{g}_{\text{Nap}} m_\infty h_p (V_m - E_{\text{Na}}) + I + I_{\text{sAHP}} + I_{\text{syn}}) + V_{\text{noise}},$$

$$\frac{dn}{dt} = \alpha_n (V_m)(1-n) - \beta_n (V_m) n, \tag{2}$$

$$\frac{dm}{dt} = \boldsymbol{\gamma_{\tau m}}(\boldsymbol{\gamma_{\alpha m}}\alpha_m (V_m - \Delta V_m)(1-m) - \boldsymbol{\gamma_{\beta m}}\beta_m (V_m - \Delta V_m) m, \tag{3}$$

$$\frac{dh}{dt} = \boldsymbol{\gamma_{\tau h}}(\boldsymbol{\gamma_{\alpha h}}\alpha_h (V_m - \Delta V_h)(1-h) - \boldsymbol{\gamma_{\beta h}}\beta_h (V_m - \Delta V_h) h, \tag{4}$$

$$\frac{dh_p}{dt} = \alpha_h (V_m)(1-h_p) - \frac{4}{1 + \exp[(V_m - V_T - 40)/5]} h_p, \tag{5}$$

where γ_{Na} modulates the maximum conductance of the sodium channels, which is analogous to altering the expression levels of the channel. $\gamma_{\tau m}$ and $\gamma_{\tau h}$ scale both rate constants α and β with the same factor so that effectively, the time constants, $\tau_i = \frac{1}{\alpha_i(V_m)+\beta_i(V_m)}$ of the $i = m$ and $i = h$ gate respectively, are scaled by $1/\gamma_{\tau i}$, while leaving the steady state, $i_\infty = \frac{\alpha_i(V_m)}{\alpha_i(V_m)+\beta_i(V_m)}$ unaffected. The kinetics of the sodium channel can also be modified by altering the rates of activation and deactivation of both the m and h gate individually using $\gamma_{\alpha m}$, $\gamma_{\alpha h}$, $\gamma_{\beta m}$ and $\gamma_{\beta h}$, leading to changes in both time constants and steady-states. The parameters ΔV_m and ΔV_h simultaneously shift the voltage sensitivity of both rate constants of the m and h gate, respectively. $\Delta V_i >$ 0 corresponds to a depolarizing shift in the voltage dependency and $\Delta V_i <$ 0 to a hyperpolarizing shift. An increase in γ_{Nap} models shifts the balance between the regular and persistent sodium current towards the persistent current. Note that γ_{Na} only increases maximal conductance of the regular sodium current. The control sodium channel model has parameter values $\gamma_{Na} = \gamma_{\tau m} = \gamma_{\tau h} = \gamma_{\alpha m} = \gamma_{\alpha h} = \gamma_{\beta m} = \gamma_{\beta h}$ = 1 and $\gamma_{Nap} = \Delta V_m = \Delta V_h$ = 0. We performed a parameter space exploration with these 10 parameters in order to map the possible effects of sodium channel modifications on the network dynamics.
